# Automatic 3D cell segmentation of fruit parenchyma tissue from X-ray micro CT images using deep learning

**DOI:** 10.1186/s13007-024-01137-y

**Published:** 2024-01-19

**Authors:** Leen Van Doorselaer, Pieter Verboven, Bart Nicolai

**Affiliations:** 1 Mechatronics, Biostatistics and Sensors (MeBioS), Biosystems Department, KU Leuven, Leuven, Belgium; 2 Flanders Centre of Postharvest Biology, Leuven, Belgium

**Keywords:** Plant microstructure, Fruit physiology, X-ray micro-computed tomography, Contrast-enhanced imaging, Image processing, Artificial intelligence, Instance segmentation

## Abstract

**Background:**

High quality 3D information of the microscopic plant tissue morphology—the spatial organization of cells and intercellular spaces in tissues—helps in understanding physiological processes in a wide variety of plants and tissues. X-ray micro-CT is a valuable tool that is becoming increasingly available in plant research to obtain 3D microstructural information of the intercellular pore space and individual pore sizes and shapes of tissues. However, individual cell morphology is difficult to retrieve from micro-CT as cells cannot be segmented properly due to negligible density differences at cell-to-cell interfaces. To address this, deep learning-based models were trained and tested to segment individual cells using X-ray micro-CT images of parenchyma tissue samples from apple and pear fruit with different cell and porosity characteristics.

**Results:**

The best segmentation model achieved an Aggregated Jaccard Index (AJI) of 0.86 and 0.73 for apple and pear tissue, respectively, which is an improvement over the current benchmark method that achieved AJIs of 0.73 and 0.67. Furthermore, the neural network was able to detect other plant tissue structures such as vascular bundles and stone cell clusters (brachysclereids), of which the latter were shown to strongly influence the spatial organization of pear cells. Based on the AJIs, apple tissue was found to be easier to segment, as the porosity and specific surface area of the pore space are higher and lower, respectively, compared to pear tissue. Moreover, samples with lower pore network connectivity, proved very difficult to segment.

**Conclusions:**

The proposed method can be used to automatically quantify 3D cell morphology of plant tissue from micro-CT instead of opting for laborious manual annotations or less accurate segmentation approaches. In case fruit tissue porosity or pore network connectivity is too low or the specific surface area of the pore space too high, native X-ray micro-CT is unable to provide proper marker points of cell outlines, and one should rely on more elaborate contrast-enhancing scan protocols.

**Supplementary Information:**

The online version contains supplementary material available at 10.1186/s13007-024-01137-y.

## Background

Many structures in plants have three-dimensional (3D) features that are difficult to infer by conventional microscopy techniques that provide two-dimensional (2D) images. Physiological processes in plants such as photosynthesis [1], respiration [2], and morphogenesis [3] are governed by transport processes of water, metabolic gasses, and nutrients that are essentially three-dimensional. These transport processes are dictated by not only shape and size of the plant and its organs, but also by the tissue morphology determined by the spatial layout of cells and intercellular space in the tissue. The 3D organization of individual cells and intercellular spaces (pores) is relevant to many physiological questions [4, 5].

X-ray micro-computed tomography (micro-CT) is a powerful imaging technique to acquire complex 3D data of plant structures. Given the tremendous advances in the development of high-resolution X-ray micro-CT laboratory instruments, this technique is becoming increasingly available to plant researchers, making them less dependent on synchrotron facilities [6, 7]. Advantages include the good penetration of X-rays into biological samples, the scalable field of view and corresponding resolution from millimeter up to micrometer and beyond. As X-ray attenuation depends on density, micro-CT is suitable to easily distinguish intercellular spaces from the cell matrix in plant tissues to explore in three dimensions the intercellular pathways for gas exchange, for example [8]. The main advantages of using micro-CT over light and electron microscopy techniques for plant imaging based on density differences, lie in its minimal requirement for sample preparation or labeling and capability to acquire detailed 3D information. Also, larger tissue samples can be imaged using X-ray micro-CT compared to microscopy imaging as the latter typically has a smaller field of view [9]. To quantify the cell morphology in tissues, the reconstructed X-ray images must be processed by a method called cell segmentation. Cell segmentation enables identification, separation and labeling of individual cells. If the cells would be completely outlined in the image, the segmentation procedure would be the easiest. Contrast-enhanced micro-CT scans can provide this by highlighting the cell walls [10, 11]. Contrast-enhancement is, however, a tedious and invasive protocol. However, in standard micro-CT scans there is not sufficient contrast in X-ray attenuation at cell-to-cell interfaces [4, 12]. Therefore, the cell outlines can only be seen at cell-to-pore interfaces rendering them incomplete. Automatic segmentation is then hard to achieve using traditional image processing pipelines, and, as a result, manual segmentation of the cell contour is often the only option that is very laborious.

Kar et al. [13] compared different deep learning (DL) pipelines for 3D cell segmentation of confocal image datasets. U-Net [14] based boundary detection models were trained to collect semantic labels, e.g., the cell centroid, cell boundary, cell matrix and background. To obtain the individual cell labels, these methods required postprocessing such as graph partitioning as was done in PlantSeg [15] or seeded watershed segmentation [16]. Kar et al. [13] reported PlantSeg as the best performing DL model compared to other pipelines. However, it should be noted that such a U-Net based boundary detection method was specifically designed for images with fluorescently labeled cell boundaries, which was similar to the data used in the comparison study. Other instance segmentation pipelines using convolutional neural networks (CNNs), first solve an object detection problem by localizing each individual object with a bounding box [17]. In Mask R-CNN, an additional step is implemented to assign a binary label to each pixel within the bounding box of a detected individual object resulting in instance segmentation. However, due to the high model complexity and long latency for object detection, the Mask R-CNN framework is continuously improved or approached differently [18, 19].

The *Cellpose* algorithm [20] was recently built to improve instance segmentation tasks compared to other state-of-the-art methods for the segmentation of a wide range of biological image data types including cells without fluorescent markers. Although *Cellpose* uses 2D data for training, the algorithm can be extended for 3D segmentation prediction by slicing a volume dataset into 2D images according to XY, XZ an YZ orientations that are recombined into 3D. In recent works, the freely available generalist *Cellpose* algorithm was mainly used on fluorescence images without retraining the model, demonstrating its excellent performance for image processing [21]. For the only application in X-ray imaging to our knowledge, *Cellpose* was used to segment the 3D structure of alveoli in intact mouse lungs of high-quality synchrotron X-ray CT data [22].

## Results

### Microstructure of parenchyma of different pome fruit cultivars

**Fig. 1:**
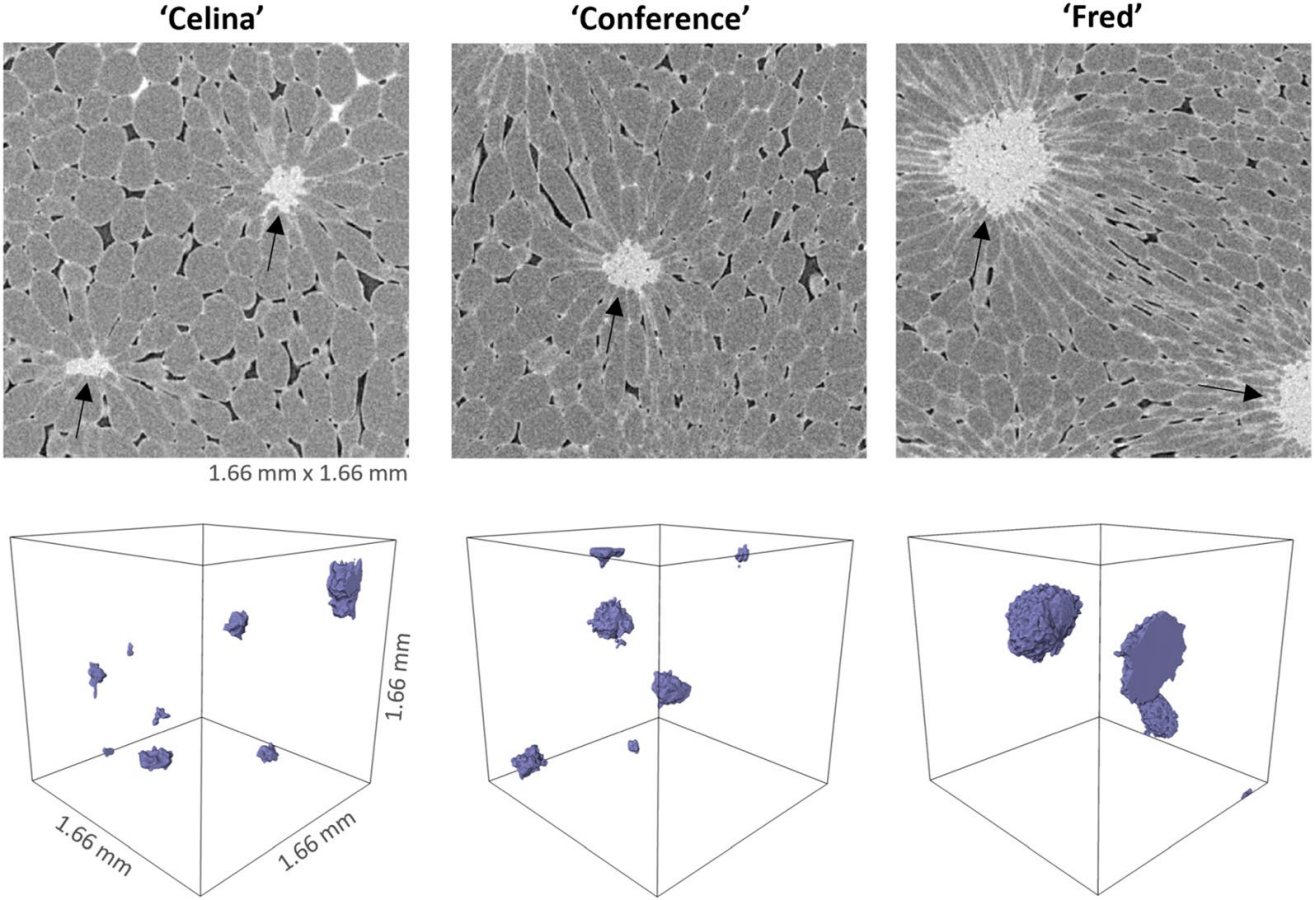
Stone cell clusters in pear tissue. (Top) 2D slices of contrast-enhanced micro-CT images of pear tissue samples of the inner cortex with stone cell clusters indicated with arrows. (Bottom) 3D visualization of the stone cell clusters

The extracted microstructural parameters of the cell matrix and pore space showed differences along the pome fruit cultivars (Table 1). Pear tissue samples contained 2.3 to 6.2 more cells and 9.1 to 46.9 times more pores than apple samples. The amount of cells and pores was higher at the inner cortex position compared to that of the outer one. The stone cell density was the highest in ‘Celina’ pears, although no differences were found for the volume fraction of the stone cells. Figure 1 shows the stone cell clusters for the three pear cultivars, which were the largest in ‘Fred’. The cell matrix anisotropy of apple tissue was higher compared to pear tissue. For the apple cultivars, the specific surface area (SSA) of the cell matrix was the lowest for ‘Braeburn’ and for pear, ‘Fred’ had a lower SSA than ‘Celina’. A similar trend was observed for the porosity, whereby ‘Braeburn’ and ‘Fred’ had the lowest for apple and pear, respectively. Overall, apple tissue had a higher porosity of 16.11–26.73% than pear tissue with a porosity of 2.98–5.09%. The Pearson correlation coefficient between pore density and porosity was − 0.33 and − 0.59 for pear and apple, respectively. Similar to pore density, the SSA of the pore space was higher in pear than apple and at the inner cortex compared to the outer cortex. The Euler number was the highest, indicating a less connected pore network, for ‘Conference’ and ‘Fred’. The Euler numbers of ‘Celina’ were the lowest and even negative for two of the three samples at both inner and middle cortex position, meaning that the pore network was a multiple connected structure in which the cell matrix was never isolated [23].

**Table 1:** Morphometric parameters of the cell matrix and pore space of three tissue samples (mean ± SD) per cortex position and pome fruit cultivar

Parameter	Cortex	‘Celina’	‘Conference’	‘Fred’	‘Braeburn’	‘Jonagold’	‘Kizuri’
Cell density [mm^−3^]	Inner	652 ± 169^Aa^	837 ± 189^Aa^	1005 ± 205^Aa^	221 ± 40^Ab^	187 ± 41^Ab^	258 ± 45^Ab^
	Middle	606 ± 75^Ba^	652 ± 103^Ba^	593 ± 193^Ba^	224 ± 10^Bb^	172 ± 32^Bb^	197 ± 49^Bb^
	Outer	743 ± 325^Ba^	626 ± 71^Ba^	695 ± 244^Ba^	183 ± 10^Bb^	162 ± 42^Bb^	215 ± 61^Bb^
Stone cell density [mm^−3^]	Inner	1.96 ± 1.43^a^	1.23 ± 0.12^b^	0.87 ± 0.00^b^	–	–	–
	Middle	2.83 ± 0.78^a^	1.16 ± 0.33^b^	1.38 ± 0.33^b^	–	–	–
	Outer	3.33 ± 1.38^a^	1.88 ± 0.45^b^	1.01 ± 0.25^b^	–	–	–
Stone cell volume fraction [%]	Inner	0.45 ± 0.50	0.42 ± 0.15	1.49 ± 1.26	–	–	–
	Middle	0.66 ± 0.56	0.72 ± 0.77	0.85 ± 0.48	–	–	–
	Outer	0.35 ± 0.14	0.45 ± 0.22	0.81 ± 0.63	–	–	–
Vascular tissue volume fraction [%]	Inner	0.21 ± 0.36	0 ± 0	0.71 ± 1.22	0.11 ± 0.11	0.08 ± 0.13	0.59 ± 0.31
	Middle	0.04 ± 0.04	0 ± 0	0 ± 0	0.05 ± 0.05	0 ± 0	0 ± 0
	Outer	1.28 ± 1.21	0.64 ± 1.06	1.64 ± 2.85	0.05 ± 0.05	0.08 ± 0.13	0.11 ± 0.20
Cell matrix anisotropy	Inner	0.007 ± 0.001^b^	0.005 ± 0.001^b^	0.008 ± 0.001^b^	0.037 ± 0.002^a^	0.036 ± 0.021^a^	0.047 ± 0.007^a^
	Middle	0.005 ± 0.002^b^	0.008 ± 0.004^b^	0.010 ± 0.002^b^	0.019 ± 0.007^a^	0.014 ± 0.009^a^	0.025 ± 0.011^a^
	Outer	0.015 ± 0.013^b^	0.007 ± 0.004^b^	0.006 ± 0.002^b^	0.022 ± 0.004^a^	0.030 ± 0.015^a^	0.028 ± 0.012^a^
Cell matrix specific surface area [mm^−1^]	Inner	15.13 ± 1.38^ab^	13.36 ± 2.48^bc^	12.78 ± 2.68^c^	13.93 ± 1.07 ^bc^	15.91 ± 1.06^a^	15.85 ± 0.56^a^
	Middle	15.27 ± 0.79^ab^	13.39 ± 1.84 ^bc^	11.45 ± 1.06^c^	14.46 ± 0.01 ^bc^	15.37 ± 0.56^a^	15.51 ± 1.29^a^
	Outer	14.74 ± 1.93^ab^	13.34 ± 1.74 ^bc^	10.07 ± 0.58^c^	13.21 ± 0.33 ^bc^	16.94 ± 1.21^a^	16.08 ± 1.28^a^
Porosity [%]	Inner	5.09 ± 1.09^c^	4.48 ± 1.27^c^	3.29 ± 0.53^d^	16.35 ± 1.47^b^	19.14 ± 1.20^a^	19.29 ± 0.98^a^
	Middle	5.05 ± 0.42^c^	4.90 ± 1.39^c^	3.87 ± 0.94^d^	16.11 ± 0.70^b^	20.84 ± 4.75^a^	22.34 ± 3.40^a^
	Outer	4.82 ± 0.18^c^	4.86 ± 1.28^c^	2.98 ± 0.54^d^	17.49 ± 0.93^b^	26.73 ± 1.13^a^	22.11 ± 2.77^a^
Pore density [mm^−3^]	Inner	2692 ± 1226^Aa^	3732 ± 495^Aa^	4784 ± 1534^Aa^	211 ± 120^Ab^	221 ± 39^Ab^	295 ± 61^Ab^
	Middle	3189 ± 646^ABa^	3322 ± 366^ABa^	2735 ± 1315^ABa^	249 ± 80^ABb^	147 ± 62^ABb^	139 ± 75^ABb^
	Outer	3271 ± 1954^Ba^	2860 ± 1092^Ba^	2995 ± 467^Ba^	138 ± 17^Bb^	102 ± 57^Bb^	171 ± 78^Bb^
Pore space anisotropy	Inner	0.12 ± 0.02^ab^	0.08 ± 0.01^ab^	0.23 ± 0.07^a^	0.16 ± 0.01^ab^	0.14 ± 0.07^b^	0.22 ± 0.04^ab^
	Middle	0.09 ± 0.04^ab^	0.16 ± 0.11^ab^	0.23 ± 0.05^a^	0.10 ± 0.04^ab^	0.08 ± 0.06^b^	0.09 ± 0.04^ab^
	Outer	0.23 ± 0.16^ab^	0.14 ± 0.09^ab^	0.19 ± 0.07^a^	0.10 ± 0.02^ab^	0.08 ± 0.04^b^	0.10 ± 0.06^ab^
Pore space specific surface area [mm^−1^]	Inner	228 ± 29^Aa^	219 ± 15^Aa^	227 ± 36^Aa^	60 ± 11^Ab^	58 ± 3^Ab^	57 ± 1^Ab^
	Middle	228 ± 17^ABa^	203 ± 27^ABa^	209 ± 27^ABa^	64 ± 4^ABb^	52 ± 13^ABb^	48 ± 9^ABb^
	Outer	231 ± 44^Ba^	203 ± 22^Ba^	225 ± 24^Ba^	52 ± 2^Bb^	42 ± 5^Bb^	50 ± 8^Bb^
Euler number	Inner	− 1202 ± 2666^c^	5761 ± 795^a^	9829 ± 2872^a^	1475 ± 646^b^	924 ± 287^bc^	1934 ± 395^b^
	Middle	− 733 ± 1076^c^	4384 ± 2609^a^	5638 ± 1877^a^	1577 ± 588^b^	728 ± 426^bc^	1015 ± 262^b^
	Outer	1274 ± 1925^c^	4654 ± 1595^a^	5939 ± 2287^a^	1023 ± 226^b^	479 ± 232^bc^	1345 ± 575^b^

Different upper case characters in the same column for each parameter indicate significant differences (p < 0.05) at different cortex position for the same cultivar. Different lower case characters in the same row indicate significant differences (p < 0.05) among different cultivars for the same cortex position

**Fig. 2:**
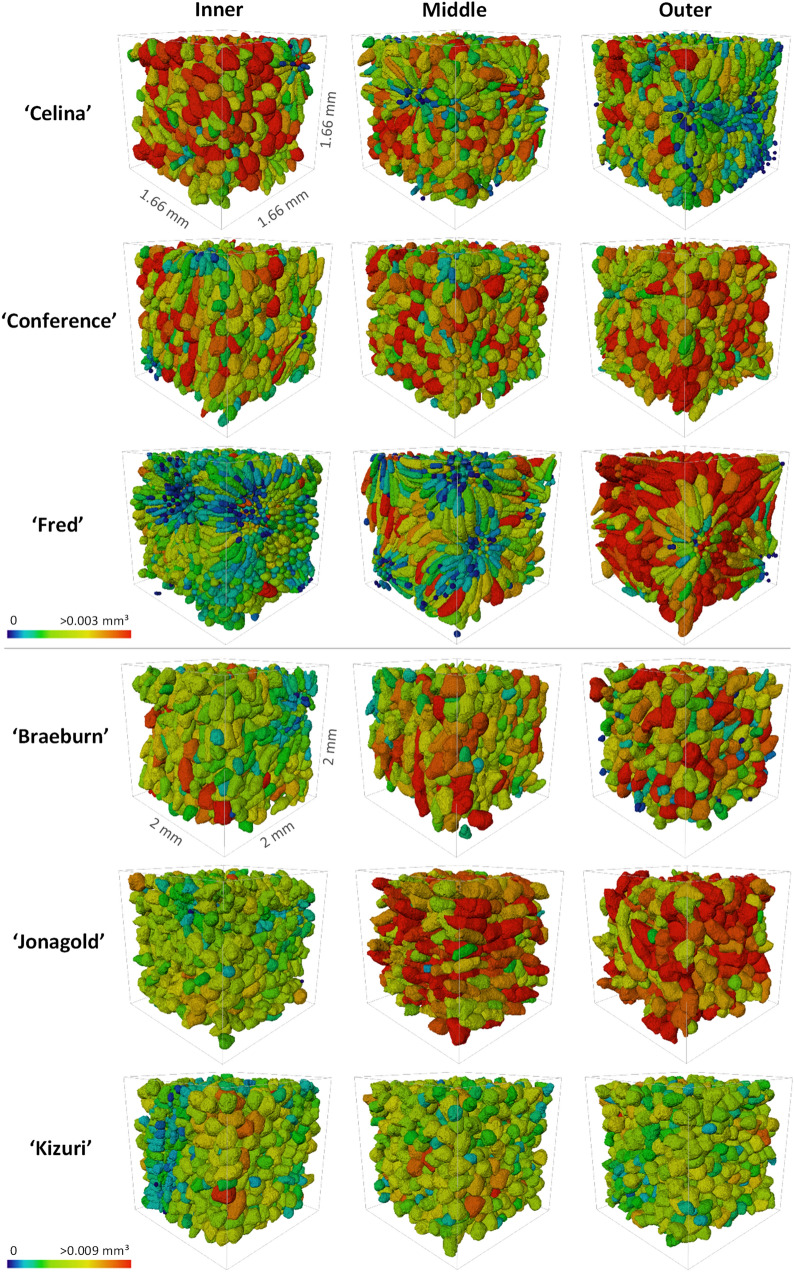
Individual cells of (top) pear and (bottom) apple cultivars at different cortex positions with labels shown in colour scale for the cell volume

**Fig. 3:**
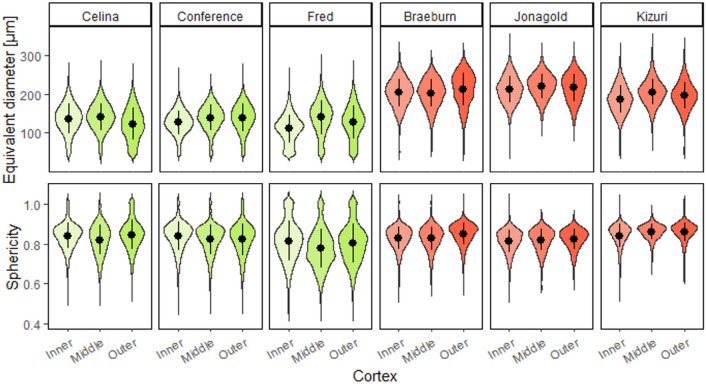
Probability density plots of the cell equivalent spherical diameter (top) and sphericity (bottom) of the pear (left) and apple (right) cultivars at different cortex positions with the dot and line representing the mean and standard deviation

After the cells were segmented from the contrast-enhanced images, the morphometric analysis was performed and results are given in Additional file 1: Table S1. The cell shape and size was different for the fruit cultivars and along the cortex position (Fig. 2). Cell length, width, volume and surface area in apple tissue were greater than in pear tissue. The cell SSA was found to be larger in pear tissue. Significant differences in cell characteristics were also found between different cultivars and different cortex positions. In Fig. 3, cell size and shape distributions are shown. For pear, ‘Fred’ had the highest fraction of small cells of all cultivars, accompanied by a wider range of cell sphericity. It was more difficult to see differences between the other two pear cultivars. For apple, ‘Jonagold’ had overall larger cells and ‘Kizuri’ smaller but more spherical cells, as also reported in Additional file 1: Table S1. Another clear observation was that the density distribution of the cell sizes in ‘Braeburn’ at the outer cortex deviated more from a normal distribution compared to the other apple tissue samples.

**Fig. 4:**
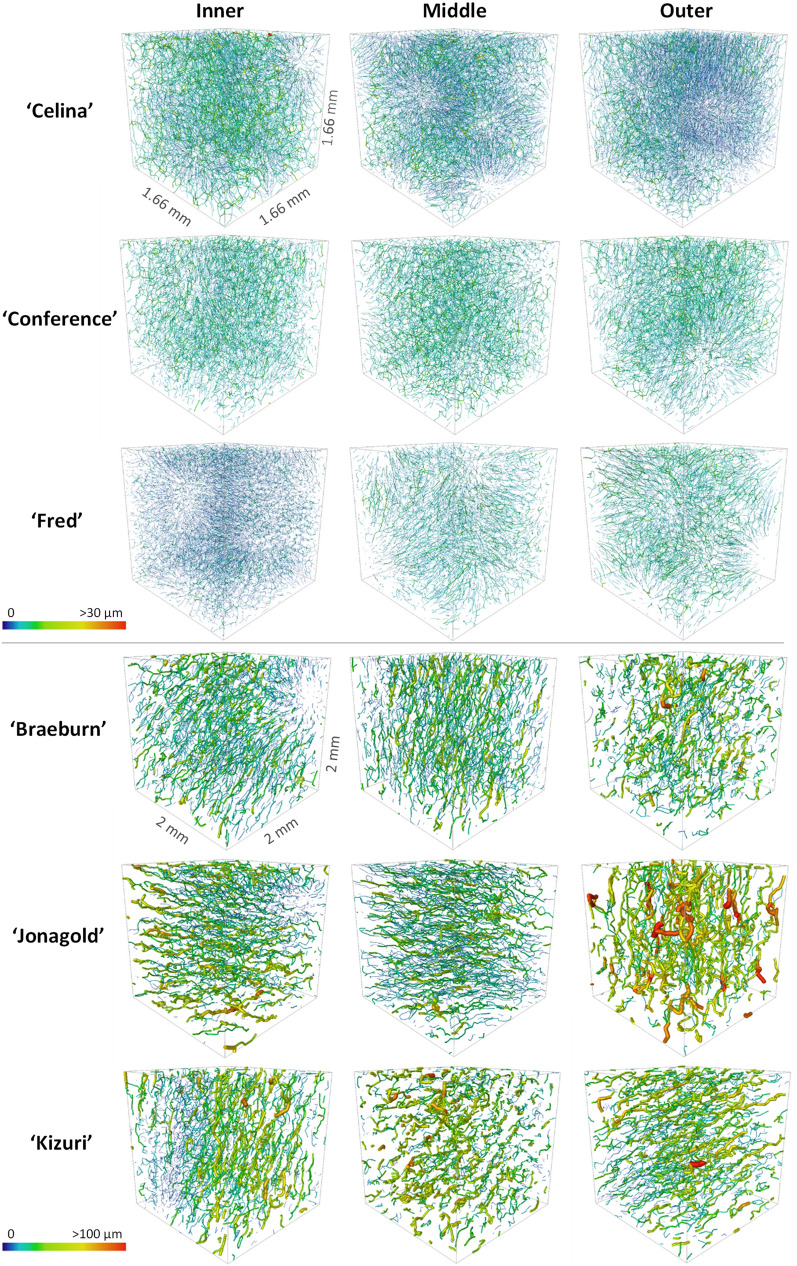
Individual pores of pear (top) and apple (bottom) cultivars at different cortex positions with labels shown in colour scale for the pore mean radius

After the pores were segmented from the conventional images, the morphometric analysis was performed and the results are given in Additional file 1: Table S2. The pore shape and size was different for the fruit cultivars and along the cortex position (Fig. 4). Pore length, width, volume and surface area in apple tissue were greater than in pear tissue. The pore SSA was found to be larger in pear tissue. Significant differences were also found between different cultivars and different cortex positions for the pore characteristics.

**Fig. 5:**
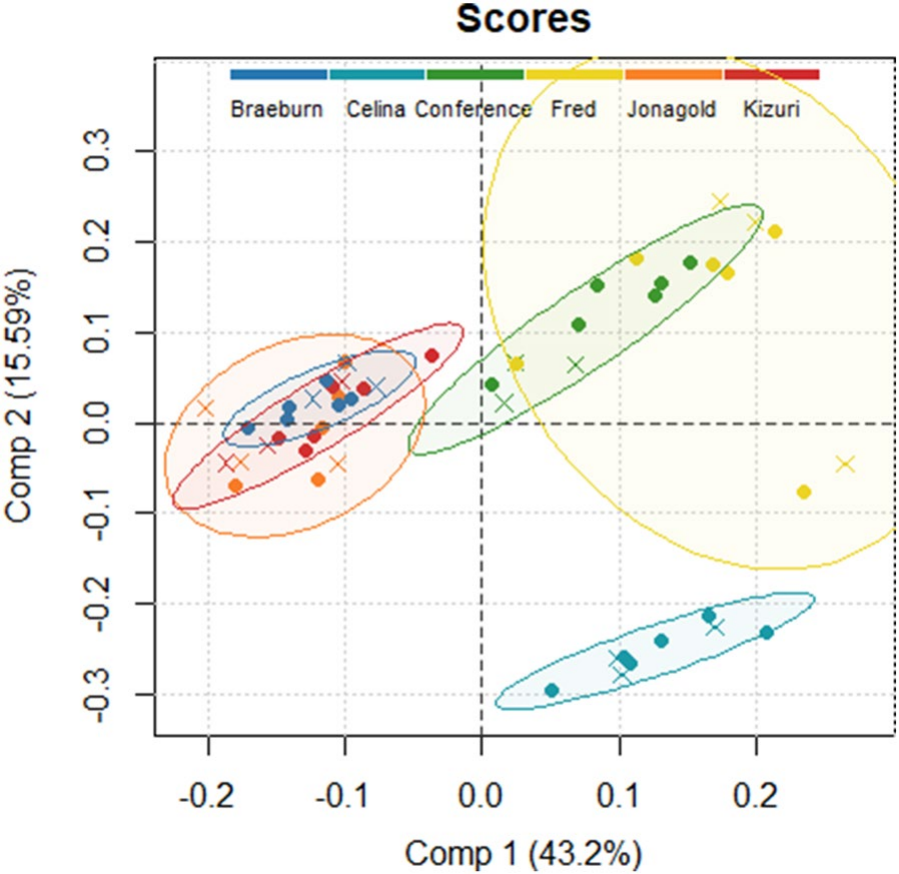
PLS-DA score plot with the scores of tissue samples used in train (•, n = 36) and test (x, n = 18) set in colour code according to the pome fruit cultivar with 95% confidence ellipses

A partial least squares discriminant analysis (PLS-DA) was applied to the morphometric and statistical parameters of parenchyma tissue with the cultivar as class label. The score plot of the first two latent variables (LVs) is presented in Fig. 5. The first two LVs explained 58.79% of the total variance in morphometric and statistical parameters. Pear cultivars are separated from the apple cultivars by the first LV, with the former having positive scores and the latter having negative scores. The SSA of the pore space had the highest importance for the first LV with a positive factor loading and was highly correlated with porosity (*r* = − 0.94), pore density (*r* = 0.96) and cell density (*r* = 0.94). Additionally, the interquartile range (IQR) of the cell SSA and pore width had a high positive and negative factor loading for the first LV, respectively. With addition of the second LV, the 95% confidence ellipse for ‘Celina’ can be separated from the other pear cultivars by lower scores. The kurtosis of the pore sphericity had the highest importance for this second LV with a negative factor loading and showed high correlation with the kurtosis of pore anisotropy (*r* = 0.91). Other LVs could not distinguish the cultivars.

### Deep learning based cell segmentation

#### Use of different slice spacings for model development

We evaluated how model training with datasets using different spacings for slice retrieval affected the final segmentation success. Table 2 summarizes the performance of the DL segmentation, expressed by the Aggregated Jaccard Index (AJI) of the test data. For both apple and pear tissue, the mean AJI slightly decreased with increasing slice spacing and thus decreasing number of training images. However, based on the statistical analysis, this improvement was much more apparent for apple tissue than for pear tissue. For apple tissue segmentation, all DL models performed significantly better than the benchmark based on the chosen evaluation metric. For pear tissue, the DL models trained with slice spacings of 128 and 512, did not perform better than the benchmark. Overall, the AJI was significantly higher for apple than for pear tissue. The mean AJI of the segmentation results by the model trained on training data retrieved with a spacing of 8 slices was 0.861 and 0.732 for apple and pear tissue, respectively. In comparison, the mean AJI of the benchmark was only 0.730 and 0.667 for apple and pear tissue, respectively. From the statistical analysis we found that the benchmark scored similarly for apple and pear tissue. For apple tissue, the model trained with a spacing of 8 slices performed best on the test data and the AJIs of the segmentation results were significantly different compared to the other DL-based segmentation models and benchmark. The same applied for pear tissue, but the models trained with spacings of 16 and 32 slices were not significantly different to the model trained with a spacing of 8 slices.

**Table 2:** Aggregated Jaccard Index (mean ± SD) of segmented cells in pome fruit tissues (n = 9) by deep learning models trained on data retrieved using different slice spacings and the benchmark method

Model	Aggregated Jaccard Index	
	Apple	Pear
8	0.861 ± 0.028^A^	0.732 ± 0.075^AB^
16	0.849 ± 0.026^B^	0.729 ± 0.081^A^
32	0.846 ± 0.027^C^	0.719 ± 0.089^BC^
64	0.842 ± 0.026^D^	0.721 ± 0.082^C^
128	0.837 ± 0.026^E^	0.718 ± 0.083^CD^
256	0.836 ± 0.029^E^	0.718 ± 0.085^C^
512	0.825 ± 0.028^F^	0.697 ± 0.092^E^
Benchmark	0.730 ± 0.034^G,ns^	0.667 ± 0.117^DE,ns^

Different upper case characters in the same column indicate significant differences (p < 0.05) between the segmentation results of models trained on pome fruit data retrieved using different slice spacings and the benchmark method using the watershed algorithm on different datasets on either apple or pear tissue samples. Between columns is marked with ‘ns’ if the model is not statistically different for apple or pear tissue

**Fig. 6:**
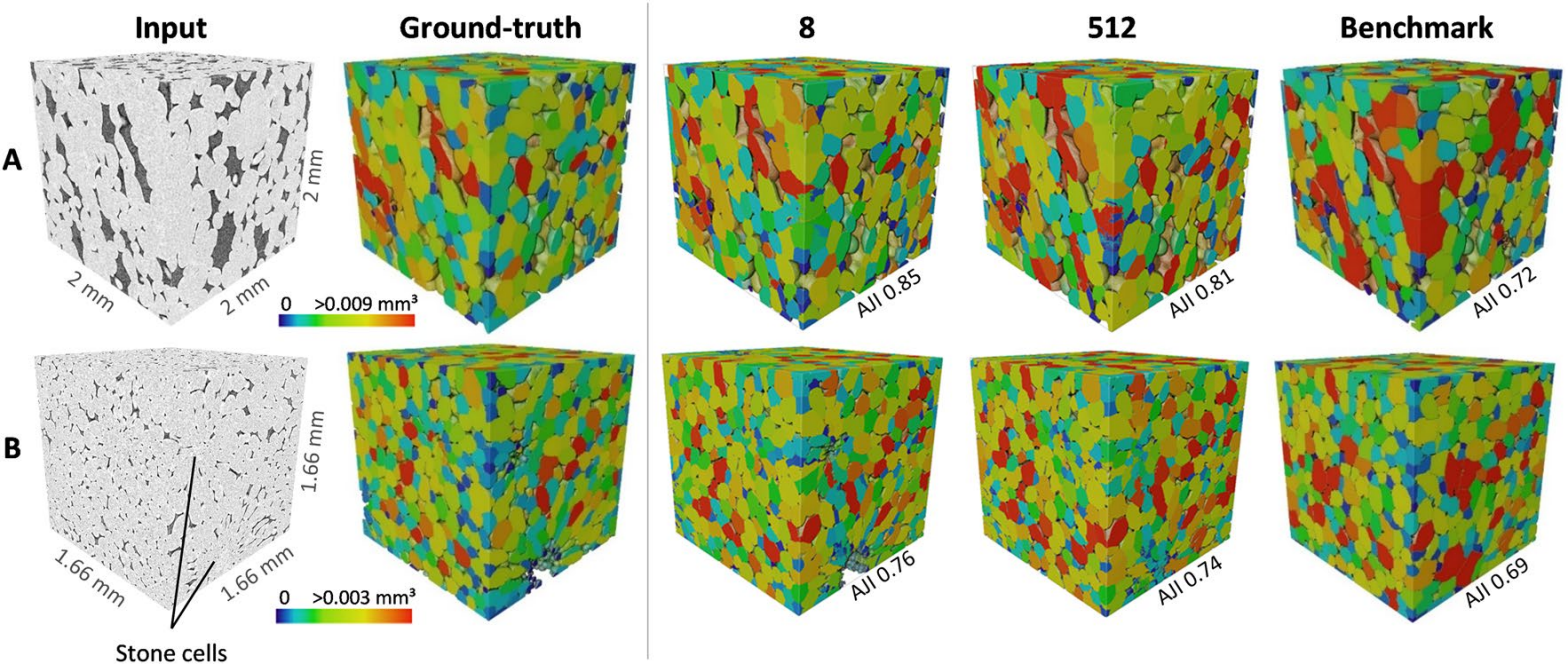
Cell segmentation results for a tissue sample of **A** ‘Jonagold’ and **B** ‘Conference’ in the test set with labels shown in colour scale for cell volume. From left to right: input volume of 667 × 667 × 667 voxels in grayscale values, ground-truth segmentation as collected with the semi-automated cell segmentation protocol, segmentation results of deep learning-based models trained with slice spacing of 8, 512 and segmentation result of the benchmark method using the watershed algorithm. *AJI* Aggregated Jaccard Index

**Fig. 7:**
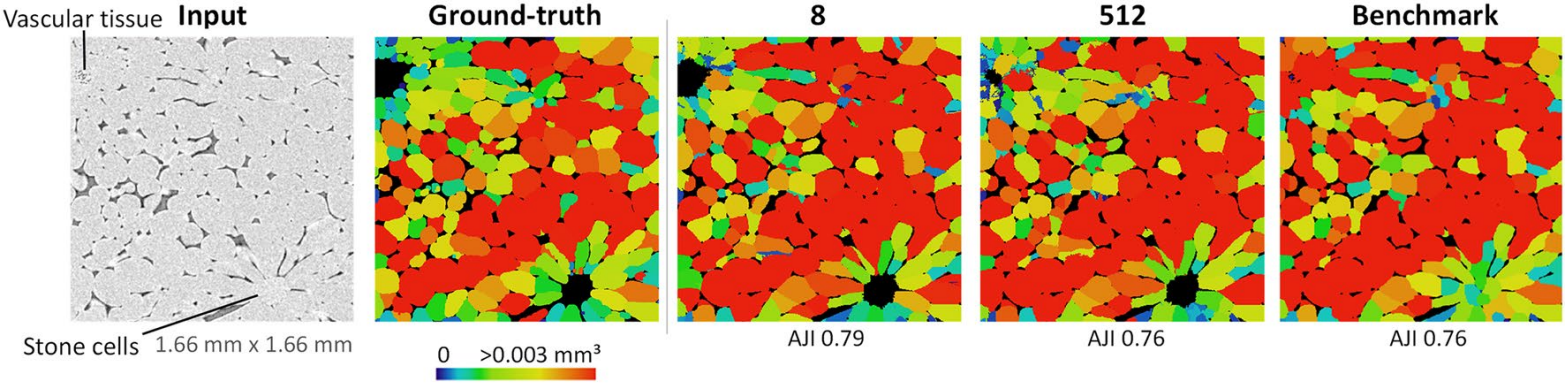
2D slice of cell segmentation results for a ‘Celina’ tissue sample with vascular tissue and a stone cell in the test set with labels shown in colour scale for cell volume. From left to right: 2D slice of the input volume of 667 × 667 × 667 voxels in grayscale values, ground-truth segmentation as collected with the semi-automated cell segmentation protocol, segmentation results of deep learning-based models trained by slice spacing 8, 512 and segmentation result of the benchmark method using the watershed algorithm. *AJI* Aggregated Jaccard Index

Figure 6 shows the segmentation results on a ‘Jonagold’ apple and ‘Conference’ pear tissue sample from the test data. The identification of clustered cells without significant intercellular spaces was problematic for the benchmark in both fruit, leading to unrealistic segmentations. Considering the DL segmentation models, the results of the model trained with a slice spacing of 8 were most similar to the ground truth (GT). For apple, the differences between the two slice spacings shown are less clear than the differences with the benchmark. For the pear sample, in which stone cells were present, the difference between the two DL-based segmentations were more apparent. Both the model trained with a larger slice spacing and the benchmark failed to properly identify the stone cell clusters. Similarly, the identification of vascular bundles was only possible with the model trained by a slice spacing of 8 (Fig. 7).

#### Transferability of deep learning models

We evaluated whether models trained on a particular fruit dataset would also perform well on different fruit, or whether data would need to be combined in training to improve performance. The resulting AJIs of the test data are summarized in Table 3. For apple tissue, the model trained on the combined dataset was significantly better than the model trained on pear data. However, the model trained on apple data solely performed better than the model trained on combined data. The original combined dataset of the second experiment comprised half the slices (5976) from both XY and YZ orientations with a slice spacing of 8. Therefore, another comparison can be made with the combined model using all slices from Table 2. This combined model and the model trained solely on apple data performed similarly for the apple tissues. For pear tissue, the model trained on the original combined dataset of 5976 slices was significantly better than the model trained on apple data (Table 3). The combined model and the model trained solely on pear data performed similarly.

**Table 3:** Aggregated Jaccard Index (mean ± SD) of segmented cells in pome fruit tissues (n = 9) by deep learning models trained on apple and pear data combined or separately and the benchmark method

Model	Aggregated Jaccard Index	
	Apple	Pear
Combined	0.849 ± 0.027^B^	0.731 ± 0.082^A^
Apple	0.863 ± 0.028^A^	0.710 ± 0.101^B^
Pear	0.821 ± 0.027^C^	0.731 ± 0.079^AB^

Different upper case characters in the same column indicate significant differences (p < 0.05) between the segmentation results of models trained on apple and pear data combined or separately on either apple or pear tissue samples. Between columns is marked with ‘ns’ if the model is not statistically different for apple or pear tissue

### Relation between microstructural features and segmentation

**Fig. 8:**
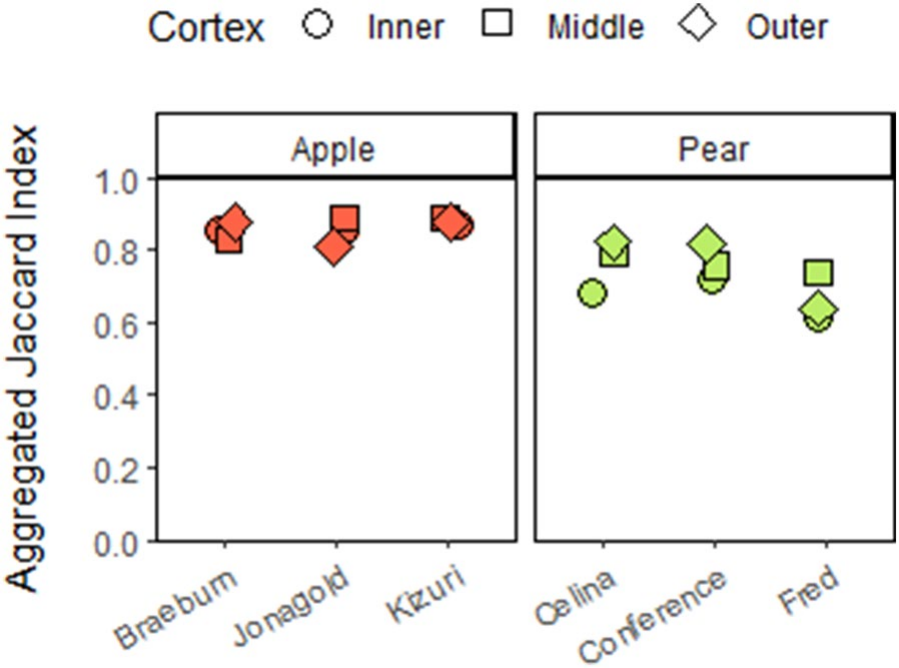
Aggregated Jaccard Index of the (left) apple and (right) pear tissue samples cell segmentations of the test set by the deep learning model trained on pome fruit data by slice spacing 8 for the different cultivars and cortex positions

**Fig. 9:**
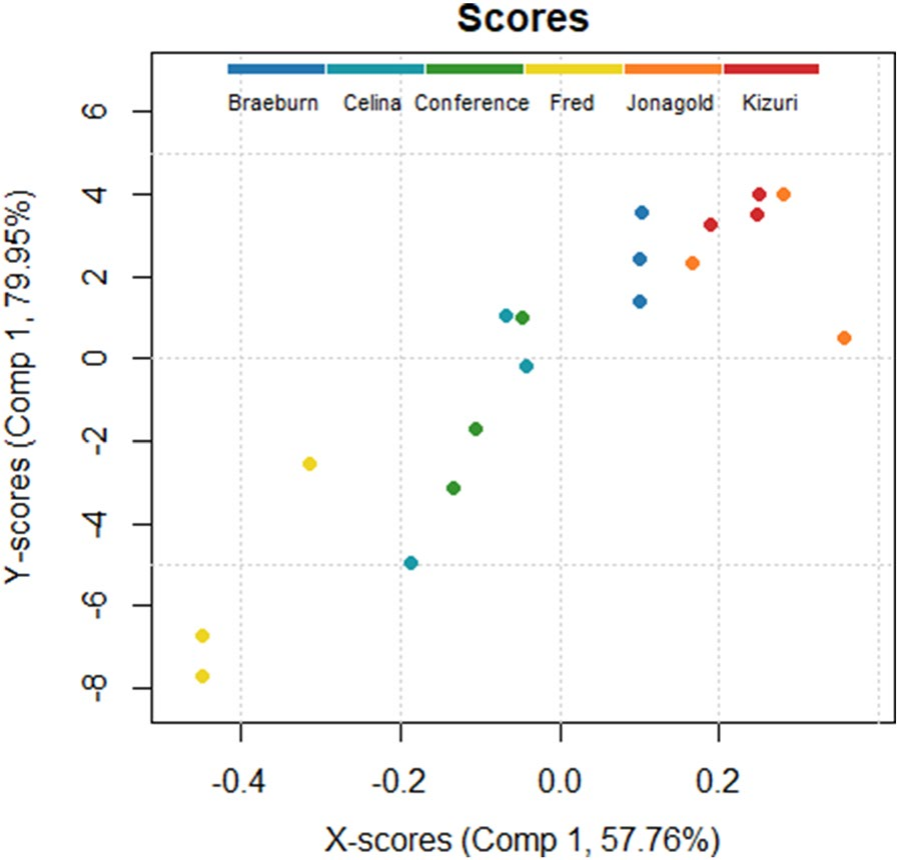
XY scores plot of the first latent variable in the PLS model to predict the segmentation results with colour code according to the pome fruit cultivar

**Fig. 10:**
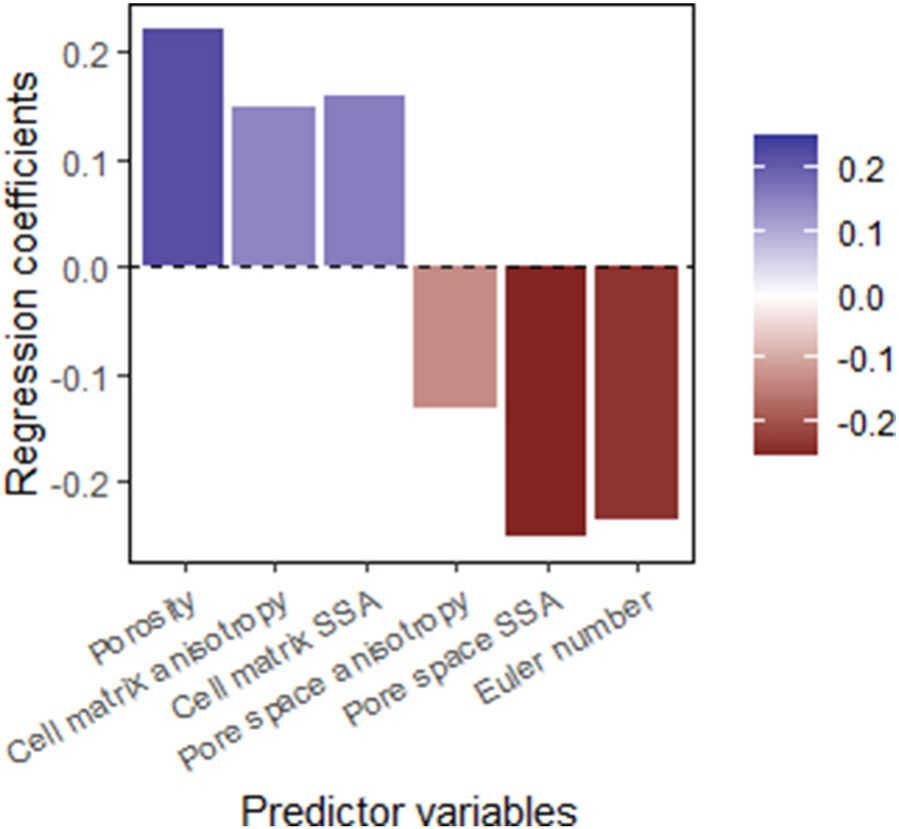
Regression coefficients of the predictor variables for the first latent variable in the PLS model, shown in colour scale, to predict the segmentation results, expressed by the Aggregated Jaccard Index. *SSA* specific surface area

The AJIs of the model trained with combined data using a spacing of 8 slices are shown for each cultivar and cortex position in Fig. 8. To explore which morphometric features of parenchyma tissue influence segmentation quality, a partial leased square regression (PLSR) analysis was performed. The PLSR model was built using the following six morphological parameters for predictor variables: porosity, cell matrix anisotropy, SSA of the cell matrix, pore space anisotropy, SSA of the pore space and Euler number. The AJIs of the model developed with combined training data retrieved with spacing by 8 slices were used as response variable. The X- and Y-scores of the first LV, which explained 57.76% of the total variance in the predictor variables and 79.95% of the total variance in the response variable, are illustrated in Fig. 9. The XY scores plot of the first LV shows a linear positive correlation and distinguishes the apple from the pear cultivars. For the first LV, all apple cultivars showed positive X-scores, while all pear cultivars negative X-scores. Moreover, ‘Fred’ and ‘Braeburn’ tissue samples had lower X-scores than the other pear and apple cultivars, respectively. Figure 10 presents the regression coefficients of the predictor variables, ranking them by their influence on the AJI, following the positive correlation found in Fig. 9. The most important predictor variable was the SSA of the pore space, which had a negative regression coefficient. The second most important and also negative predictor variable was the Euler number. For a sample with a lower Euler number, meaning increased connectivity of the pore network, the PLSR model predicts a higher AJI. The third most important predictor variable was the porosity, which had a positive regression coefficient.

## Discussion

### Deep learning-based instance segmentation outperforms watershed segmentation

Automatic instance segmentation of 3D X-ray micro-CT data of plant tissue was achieved using a DL-based model on the freely available *Cellpose* network [20]. Unlike other DL approaches where the focus is mainly on collecting semantic labels, the 2D U-Net of *Cellpose* was trained on transformed image representations of directional heat diffusion within a cell. By combining the predictions in XY, YZ and XZ orientations, the 3D organization of individual cells was obtained. The approach outperformed the current state-of-the-art method for cell segmentation of plant tissue micro-CT images with the marker-based watershed algorithm. This can be explained for multiple reasons. First, the binary image of the cell matrix, collected using Otsu’s threshold, contains vascular tissue in addition to cells. Moreover, brachysclereids (stone cells; clusters of small cells with thick lignified cell walls) that appear frequently in pear tissue are also present. As a result, the vasculature and stone cells will be segmented as if they were cells using the benchmark method (Fig. 7). Second, when the cells are segmented with a marker-based watershed algorithm, the marker extent parameter is typically chosen based on visual inspection. However, the segmentation is highly sensitive to changes in this marker, provoking non-optimal segmentations for different samples due to the highly heterogenous tissue structure (Additional file 2). Third, the watershed algorithm cannot properly segment densely clustered cells as was already reported by Herremans et al. [5].

### Deep learning training requires data that retain spatial correlations

It was overall found that the smaller the interspacing between training data slices, the better the segmentation model. This can be for several reasons. First, pear tissue contains stone cell clusters which ensure a very compact arrangement of cells (Fig. 1). Second, apple parenchyma tissue exhibited a significant anisotropy, with cells and intercellular spaces having preferred radial orientation [8]. Finally, the vascular bundles can be better detected by models trained with lower slice spacing. All these features have a short distance spatial correlation that needs to be resolved in the training. Loss of correlation by using more distant 2D slices makes proper segmentation of these features difficult or impossible.

### Deep learning training with more diverse data works best for segmenting all tissue types

It was demonstrated that a generalist (i.e., combined) model performs similarly to the specialist models, given that the *Cellpose* model obtains a representation of the image style of each [20]. This allows the model to learn how to handle different styles differently, which is interesting for further applications where one can think of extending the model to segment new pome fruit cultivars or other plant tissues that have different microstructures. Still, the combined model requires more data to train. Transferring the model from one fruit to the other was not successful, so future extensions likely will require retraining.

### Improving accuracy and generalizability in future work

One of the advantages of a 2D model is that even with a limited amount of labeled 3D samples, thousands of images could be generated for the training set. However, a 3D model may be able to achieve better results and increase the generalizability, especially in the area around stone cell clusters and vasculature. In 2022, Eschweiler et al. [24] developed a 3D model to extend the *Cellpose* approach to improve segmentation accuracy on 3D data. Furthermore, they simplified the computation of complex gradient maps by using a hyperbolic tangent spanning, while still achieving similar segmentation accuracy. Future research will have to show whether such 3D models can outperform the current 2D models and further improve cell segmentation quality.

### Segmentation quality can be linked to difference in microstructure

Considerable differences and changes in microstructure of parenchyma tissue of different fruit cultivars along the cortex position were revealed as summarized in Table 1, Additional file 1: Tables S1 and S2, and shown in Fig. 3. The PLS-DA visualizes the structure of the dataset (Fig. 5) and identifies crucial microstructural features to distinguish pome fruit cultivars. The first LV succeeded to differentiate pear from apple tissue, while the second LV was able to separate ‘Celina’ from the other cultivars. After selecting the best segmentation model, we tried to verify if the segmentation quality can be explained by these differences in microstructure. Hereto, a PLSR analysis was performed with only using the morphological parameters that can be obtained from conventional X-ray micro-CT images of fruit tissue (Fig. 9). The first LV of the PLSR model also succeeded to differentiate pear from apple tissue and additionally was able to distinguish ‘Fred’ and ‘Braeburn’ from the other pear and apple cultivars, respectively.

The SSA of the pore space, highly negatively correlated to porosity, was the most important morphometric parameter to predict the segmentation quality (Figs. 9 and 10), both of which were also relevant in the PLS-DA. Pear is known to be less porous than apple. Moreover, ‘Braeburn’ and ‘Fred’ emerged as the least porous apple and pear cultivars analysed in this study (Table 1). Additionally, the SSA of the pore space was higher in pear tissue compared to apple. This means that for the same amount of pore volume, pear has more cell-pore interfaces which can explain the difference in layout of the cells and pores between apple and pear fruit: pear cells are more homogeneously distributed within the fruit tissue where almost all cells are surrounded by small connected pores. On the other hand, apple tissue has large pores and the cells are typically more clustered compared to in pear. For this reason, other studies suggested that pore formation in pear occurs in a schizogenous manner, while in apple pores would be formed in a lysigenous manner [8, 25]. The observation that the segmentation models work better on apple tissue compared to pear can thus be explained by the difference in porosity or SSA of the pore space. In fact, despite the high correlation of the SSA of the pore space with the porosity, the first might still have added value in predicting the segmentation quality as it was found to be typically higher in the inner cortex tissue compared to outer (Table 1). In Fig. 8 can be seen that the inner cortex tissues in pear typically had a lower AJI. Given that this could not be determined for apple, this would indicate that for low porous pear tissue, an increase in SSA of the pore space has a more detrimental effect on the segmentation than for apple. Similarly, from Figs. 8 and 9 it is clear that the segmentation quality was lower for ‘Fred’ and not for ‘Braeburn’ compared to the other pear and apple cultivars, respectively. For the low porous pear tissue, a further decrease in porosity is more detrimental than in apple, indicating porosity as a limiting factor. Additionally, a strong correlation with cell and pore density suggests that segmentation becomes more difficult when cells are densely packed, which is typical for pear and/or inner cortex tissue (Table 1). Both apple and pear had a negative Pearson correlation coefficient between porosity and pore density, which was weaker for pear. However, for ‘Braeburn’ and ‘Fred’, pore density was similar to that of the other apple and pear cultivars, while porosity was lower (Table 1). An explanation could be found in the large variability in the number of pores per tissue sample, mainly caused by the presence or absence of vasculature, which is reflected in the relatively high standard deviations in pore density. Similarly, the weaker negative correlation found in pear could be explained by different density, shape and size of stone cell clusters, which greatly affect the microstructure in pear tissue. This was also reflected in the difference in standard deviation in pore density between apple and pear tissue, which was 84 and 1133/mm^3^, respectively. Finally, in the PLS-DA, the IQR of the cell SSA and pore width also had a high importance for the first LV. More specifically, the distribution of the cell SSA is more spread out in pear cortex tissue compared to apple, likely due to the presence of stone cell clusters. These clusters greatly contribute to a variety of cell shapes (Fig. 2, top), as the surrounding cells are typically elongated in the radial direction (Fig. 1, top). Additional file 1: Table S1 indeed showed that 1.7–4 times higher standard deviations of cell SSA were found for pear. ‘Fred’ typically had the highest standard deviation, which can be explained by the large stone cell clusters that have a greater impact on the layout of the cells (Figs. 1 and 2). Meanwhile, the distribution of the pore width would be more uniform in pear. Additional file 1: Table S2 showed that 2.9–8.3 times smaller standard deviations of pore width were found. This is again probably due to schizogenous pore formation in pear tissue where pores are formed by cell separation during growth opposed to lysigenous pore formation in apple caused by cell death.

The Euler number was the second most important morphometric parameter to predict the segmentation quality. For a sample with a lower Euler number, meaning increased connectivity of the pore network, the PLSR model predicts a higher AJI. ‘Celina’ could be distinguished from the other pome fruit cultivars based on the second LV of the PLS-DA, due to its lowest Euler numbers (Fig. 5 and Table 1). The highest Euler numbers, meaning reduced connectivity, were found in ‘Conference’ and ‘Fred’ tissue. Probably ‘Celina’ had increased connectivity, especially compared to ‘Fred’ and ‘Conference’, because of the smaller and more frequent stone cell clusters (Fig. 1 and Table 1). In the PLS-DA, the kurtosis of the pore sphericity, highly related to kurtosis of pore anisotropy, had the highest importance for the second LV. Meaning that in ‘Celina’ tissue the distribution of both pore shape characteristics typically had a high peak with heavy tails, indicating outliers. Again, this can be explained by the more homogeneous cell matrix related to the frequent presence of small stone cells in ‘Celina’ that affect the pore network and connectivity differently compared to in ‘Fred’ and ‘Conference’.

The developed DL-based model can be used not only for cell segmentation and analysis within fruit, but also between fruit of the same cultivar or between fruit of different cultivars. For future application of the model to segment parenchyma tissue, the porosity, SSA of the pore space and Euler number can be easily determined. From these, a rough estimation can then be made as to whether these tissues can be segmented with the same quality as determined in this study. For example, a less porous pear tissue than ‘Fred’ would probably result in poor segmentation quality and misleading results. In addition, Fig. 1 illustrated the biological variability in stone cell clusters in pear, which greatly affect the microstructure. This could hinder the use of the DL-based segmentation model for new pear cultivars. On the other hand, the model would be better suited to segment new apple cultivars as the microstructure is less heterogeneous compared to pear.

### Microstructure might explain susceptibility to physiological disorders

Apple cultivar ‘Braeburn’ is known to be highly susceptible to physiological disorders induced by CA [26–28]. Additionally, ‘Celina’ and ‘Fred’ are difficult pear cultivars to store over a long storage period (VCBT, personal communication). ‘Celina’ is very sensitive to CO_2_ and best stored in storage conditions below 0.5% CO_2_.

Previous studies using multiscale modelling reported that the lowest O_2_ concentrations were found within apple [2] and pear cells [32] due to respiration and gas diffusion at the microscale level within the fruit. Therefore, resolving the spatial organization of the cells is essential for the detection of the lowest O_2_ concentration within the fruit to prevent the development of anoxia-related physiological disorders during CA storage [33].

For ‘Fred’ and ‘Braeburn’, this increased susceptibility might be explained by the porosity as it was the lowest in the pear and apple cultivars analysed in this study, respectively (Table 1). As low porosity increases the chance of disconnected pores and limits gas diffusion, local anoxic or hypoxic conditions may occur inside the fruit which can initiate the development of physiological disorders [29, 30]. Additionally, it was found that the SSA of the cell matrix was the lowest in ‘Braeburn’, for all apple cultivars, and lower in ‘Fred’ compared to ‘Celina’. This implies that for the same volume of cell matrix, more clustering occurs. As clustering decreases the surface for gas-fluid exchange, the overall gas conductance is reduced as gas diffusivity in water is lower than in air [2].

‘Jonagold’ has been shown to have higher gas diffusivity and permeability of cortex tissue than ‘Braeburn’ [36], which is in line with the observed lower porosity and SSA of the cell matrix of the latter (Table 1). ‘Braeburn’ has a larger respiration rate, potentially aligning with a higher cell density compared to ‘Jonagold’. Differences in cell density were not significantly different in this study, but the above correlates well with the smaller cell sizes found in ‘Braeburn’ (Fig. 3 and Additional file 1: Table S1.).

Low SSA of the cell matrix, and thus high clustering, in ‘Fred’ can be explained by looking at organisation, shape and size of the cells around the large stone cell clusters in Fig. 1. The smaller and more frequent stone cell clusters in ‘Celina’ likely clarify the lower clustering compared to ‘Fred’ (Table 1). In addition, ‘Fred’ had more small cells compared to the other pear cultivars (Fig. 3). In mango fruit, it was observed that the respiration rate during ripening was related to cell density and inversely related to cell size [31]. Consequently, this could support the hypothesis that higher respiration occurs because of the higher amount of small cells within ‘Fred’ pears.

Despite the fact that ‘Celina’ had a well-connected pore network, in practice this cultivar is found to be very sensitive to physiological disorders (unpublished data). However, only a few differences in cell and pore morphology according to the cortex position compared with the other pear cultivars were found (Additional file 1: Tables S1 and S2). The main observations for ‘Celina’ were that the stone cell density was remarkably higher and that more small cells were found in the outer cortex (Fig. 3). The significant presence of stone cell clusters likely hinder O_2_ and CO_2_ diffusion by altering the structure of the pore network. This obstructed gas diffusivity may contribute to typical gas conditions in the fruit that are related to the development of physiological disorders [30, 34, 35]. Additionally, stone cell clusters could worsen the creation of unfavourable gas conditions in the fruit due to their impact on cellular gas transport. Ho et al. (2011) hypothesized that although O_2_ and CO_2_ diffuse faster in air than in water, the higher solubility of CO_2_ in water would indicate that cells provide an additional transport route for CO_2_, which was supported by their computational results. Therefore, CO_2_ diffusion through the cell matrix is probably hindered by the stone cell clusters or at least may affect the intracellular CO_2_ distribution. The same reasoning can be made for ‘Fred’, which had remarkably large stone cell clusters (Fig. 1). Additionally, the higher amount of small cells in the outer cortex region of ‘Celina’ could also affect gas distributions through locally increased respiration (Fig. 3). Altogether, this could contribute to unfavourable gas conditions initiating the development of physiological disorders.

## Conclusion

A DL-based method to obtain the complete 3D tissue microstructural information of pome fruit from X-ray micro-CT imaging of tissue samples was developed. The *Cellpose* model was adopted to perform instance segmentation without the need for contrast enhancement and manual segmentation steps. Two experiments were conducted to determine the best segmentation model. There appeared to be a trend that the more data, and thus lower slice spacing to retrieve 2D training images, the better. A generalist pome fruit segmentation model performed as well as the specialist models of apple and pear tissue, separately.

For the relation between the microstructural features and segmentation, the lower SSA of the pore space and higher porosity, which are typical for apple tissue, promote the segmentation quality. Furthermore, a high Euler number, indicating lower connectivity of the pore network, would worsen the segmentation. Substantial differences in the microstructure of the parenchyma tissue of different fruit cultivars along the cortex position were found which might explain susceptibility to physiological disorders. Notable was the effect of the size of stone cell clusters in pear on the shape, size and orientation of neighbouring cells.

In the future, the segmentation model will be extended to 3D to further improve segmentation quality. In addition, vascular tissue and stone cells will be segmented separately, as it could already be established in this study that a neural network is able to recognize them.

## Methods

The aim of this work was to train and test a DL network for 3D plant tissue segmentation into individual cells from X-ray micro-CT images without a need for contrast-enhancing and manual segmentation steps. Parenchyma tissue samples from different pome fruit were used for imaging and analysis, for the following reasons:

- Parenchyma tissue is abundant and easy to sample in the hypanthium of mature apple and pear fruit. Parenchyma consists of cells and pores with wide distributions of size and shape providing large and variable datasets [37–39].
- Apple and pear fruit parenchyma are distinct as the cells and pore characteristics have been shown to be significantly different, even between cultivars [5, 40]. This allows to test generalizability and transferability of the results.
- The storability of pome fruit, and hence their availability to consumers throughout the year, depends heavily on their gas exchange properties [1]. Unravelling the complex cellular architecture helps in understanding the susceptibility to physiological disorders.
- A benchmark segmentation pipeline is available using conventional image processing with the marker-based watershed algorithm [5].

To achieve the objective, the *Cellpose* model was adopted to develop instance segmentation algorithms for accurate and automated cell segmentation of tissue micro-CT images. First, an X-ray imaging protocol was developed and applied to perform conventional and successive contrast-enhanced scans on the same tissue samples. Then, the corresponding images were registered and a semi-automated segmentation workflow was developed and validated to extract cell labels from the contrast-enhanced scans as GT data. *Cellpose* was trained on the 2D slices of the conventional micro-CT images against the GT labels. Training based on different numbers of images was investigated. Further, the DL model was trained on the images of apple or pear parenchyma tissue separately or combined to test transferability to different datasets. The different models were then evaluated and compared to the benchmark cell segmentation method. Finally, the relationships between microstructural parameters and segmentation quality were investigated to understand why the performance may be different on different microstructures.

### Pome fruit

Commercial pear (*Pyrus communis* L.) cultivars ‘Celina’, ‘Conference’ and ‘Fred’ were harvested by a Flemish grower (50°53′48.80″ N 5°08′48.25″ E, Geetbets, Belgium) on August 23, September 11 and 22, 2021, respectively. Commercial apple (*Malus* × *domestica* Borkh.) cultivars ‘Jonagold’, ‘Kizuri’ and ‘Braeburn’ were harvested by another Flemish grower (50°49′4.10″ N 4°46′34.66″ E, Bierbeek, Belgium) on October 7, 19 and 26, respectively. The fruit were stored for several days to no more than a month after harvest until used for X-ray imaging. ‘Fred’ and ‘Celina’ were stored under regular air at 0 °C and ‘Conference’ at − 1 °C. ‘Braeburn’ and ‘Kizuri’ were stored under regular air at 1 °C and ‘Jonagold’ under controlled atmosphere conditions (1% O_2_, 3% CO_2_) at 1 °C.

### X-ray micro-CT

**Fig. 11:**
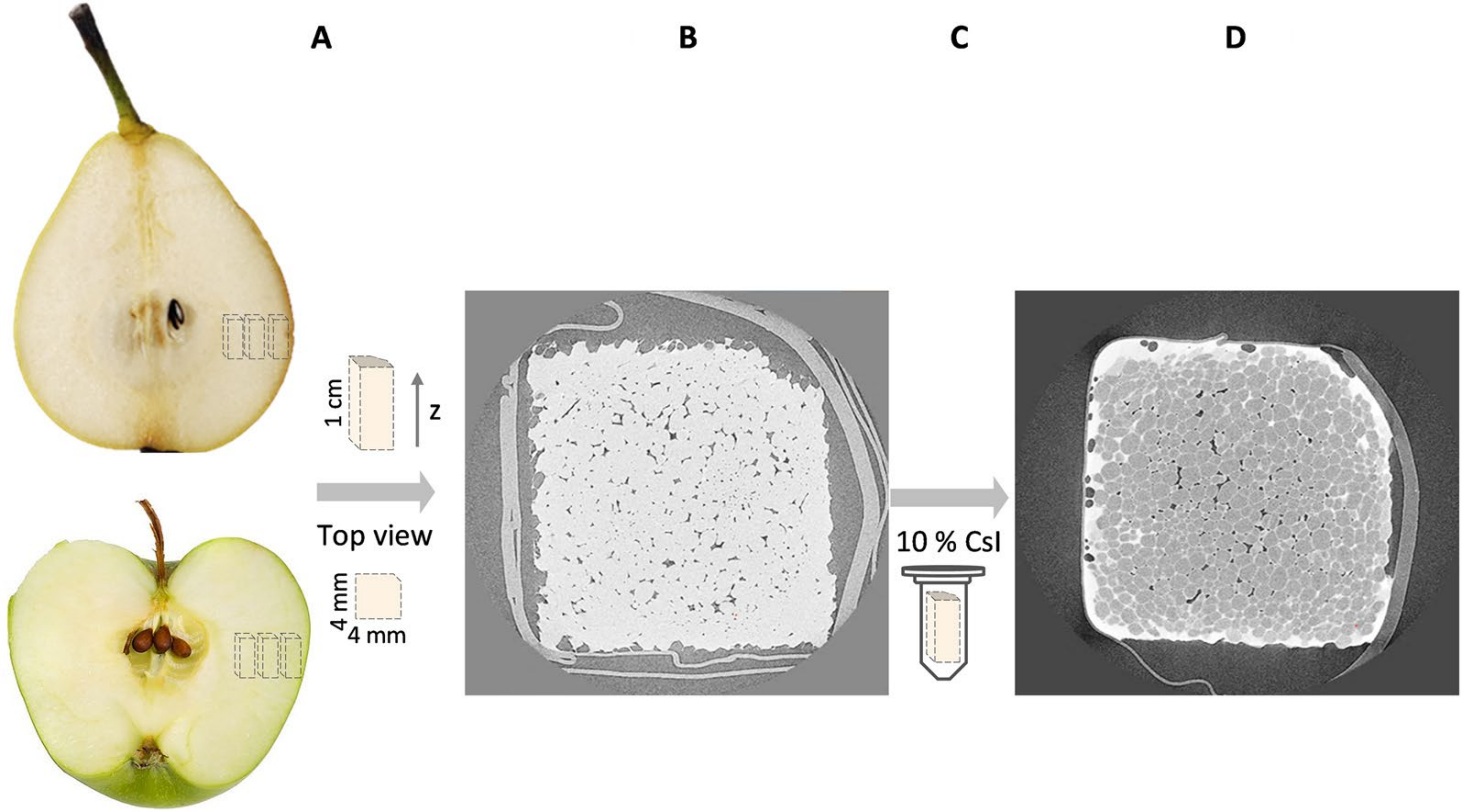
**A** Excision of pome fruit parenchyma tissue samples at inner, middle and outer positions on the fruit equator followed by **B** conventional X-ray micro-CT imaging. **C** Incubation of the tissue sample in a 10% (w/v) cesium iodide solution **D** followed by contrast-enhanced X-ray micro-CT imaging

The X-ray imaging protocol to perform conventional and successive contrast-enhanced scans on the same tissue samples is illustrated in Fig. 11. Sampling of the pome fruit tissue was done in a standardized manner (Fig. 11A). Inner, middle and outer cortex tissue samples of 4 × 4 × 10 mm were excised with a razor blade from the fruit equator at radial positions 0.6 *R*, 0.75 *R* and 0.9 *R*, with *R* the pome fruit radius, respectively. A corner piece was cut off, the sample was patted dry with tissue paper and then wrapped in parafilm to prevent dehydration while scanning. X-ray projection images of the tissue sample were acquired using a UniTom HR micro-CT system (Tescan XRE nv, Ghent, Belgium) with voxel resolution of 3 µm at 60 kV and 5 W for apple and 2.5 µm at 55 kV and 6 W for pear (Fig. 11B). Two thousand projection images were captured with an exposure time of 140 and 310 ms for apple and pear tissue samples, respectively. After performing the conventional scan, the sample was carefully unwrapped and incubated in a 10% (w/v) caesium iodide solution for 1 (all pear cultivars and ‘Jonagold’) or 2 h (‘Braeburn’ and ‘Kizuri’) while agitating every 15 min (Fig. 11C). After incubation, the above-mentioned scanning procedure was repeated to obtain a contrast-enhanced scan (Fig. 11D). The projection images of both scans were reconstructed using the filtered back-projection method in Panthera (Tescan XRE nv, Ghent, Belgium). For every apple and pear cultivar, four fruit were sampled at the three cortex positions. However, some scans were unusable due to motion artifacts during micro-CT imaging resulting from damage done by the contrast-enhancement procedure. As a result, three repetitions per sampling position were finally used, which provided nine samples per cultivar.

### Image registration

The reconstructed 16-bit grayscale image stacks were converted to 8-bit using MATLAB R2020b (The Mathworks, Inc., Natick, MA, USA) and the following image processing protocol was performed in Avizo 2020.3.1 (Thermo Fisher Scientific, Waltham, MA, USA). First, the corresponding image stacks of the same tissue sample were registered (aligned in 3D), as illustrated in Additional file 3. The samples were manually pre-aligned to reduce computational time. The inner part of the tissue sample was indicated as a rectangular subsample to perform the image registration with rigid and aniso-scale transformation using the normalized mutual information metric based on the publication of Studholme et al. [41]. Following the image registration, a volume of interest (VOI) of 667 × 667 × 667 voxels was extracted at the same position for both scans.

### Cell matrix and pore labelling

The conventional images were used to extract the binary volume of the cell matrix, the pore space and the individual pore labels as illustrated in Additional file 4. Hereto, the cell matrix was segmented using Otsu’s thresholding [42] after a filtering step. The binary of the cell matrix was denoised using a morphological opening operation. After that, the binary of the cell matrix was inverted to retrieve the pore space. Next, watershed segmentation was applied to obtain the individual pore labels after determining the optimal segmentation marker using the highest Calinski-Harabasz index value in MATLAB [43].

### Ground truth cell labels

A semi-automated cell segmentation workflow was developed to extract individual cell labels from the contrast-enhanced scans that will serve as GT (Additional file 5: Fig. S1). The workflow consisted of two steps. First, voxels with grayscale values higher than those of the cells, i.e., cell walls, pores filled with contrast agent, vascular tissue and brachysclereids (stone cells; clusters of small cells with thick lignified cell walls) were removed to mask only cells and some remaining cell walls. Second, these remaining cell walls were removed from the cell matrix by setting a manual threshold before performing watershed segmentation. The segmented cells were labelled and dilated to compensate for previous removal of the cell wall. The vascular tissue, if present, was manually segmented over multiple 2D slices in XY orientation using a region growing algorithm and interpolated to retrieve the 3D segmentation. The 3D segmentation was checked and refined in the YZ and XZ orientation. The same applied to stone cells that appear frequently in pear tissue. The semi-automated cell segmentation workflow for collecting GT cell labels was validated by comparison with a manually corrected dataset, as reported in Additional file 5.

### Morphometric parameters of tissues from different pome fruit cultivars

The cell matrix, pore space, pore labels and cell labels were analysed to obtain characteristic morphometric parameters. The calculated morphometric parameters at the sample level (cell matrix and pore space) and for individual cells and pores are described in Additional file 6. The mode, IQR, skewness and kurtosis were extracted as statistical parameters for all cell and pore parameter distributions. A total of 83 parameters were collected per sample. A PLS-DA with full cross-validation was performed on standardized data to visualize the structure of the dataset with different cultivars using mdatools [44] in RStudio 1.2.5033 (RStudio Inc., Boston, MA, USA). The loadings of the first two LVs were evaluated to identify key microstructural features to differentiate between different pome fruit cultivars.

### Dataset generation for deep learning model training and testing

**Fig. 12:**
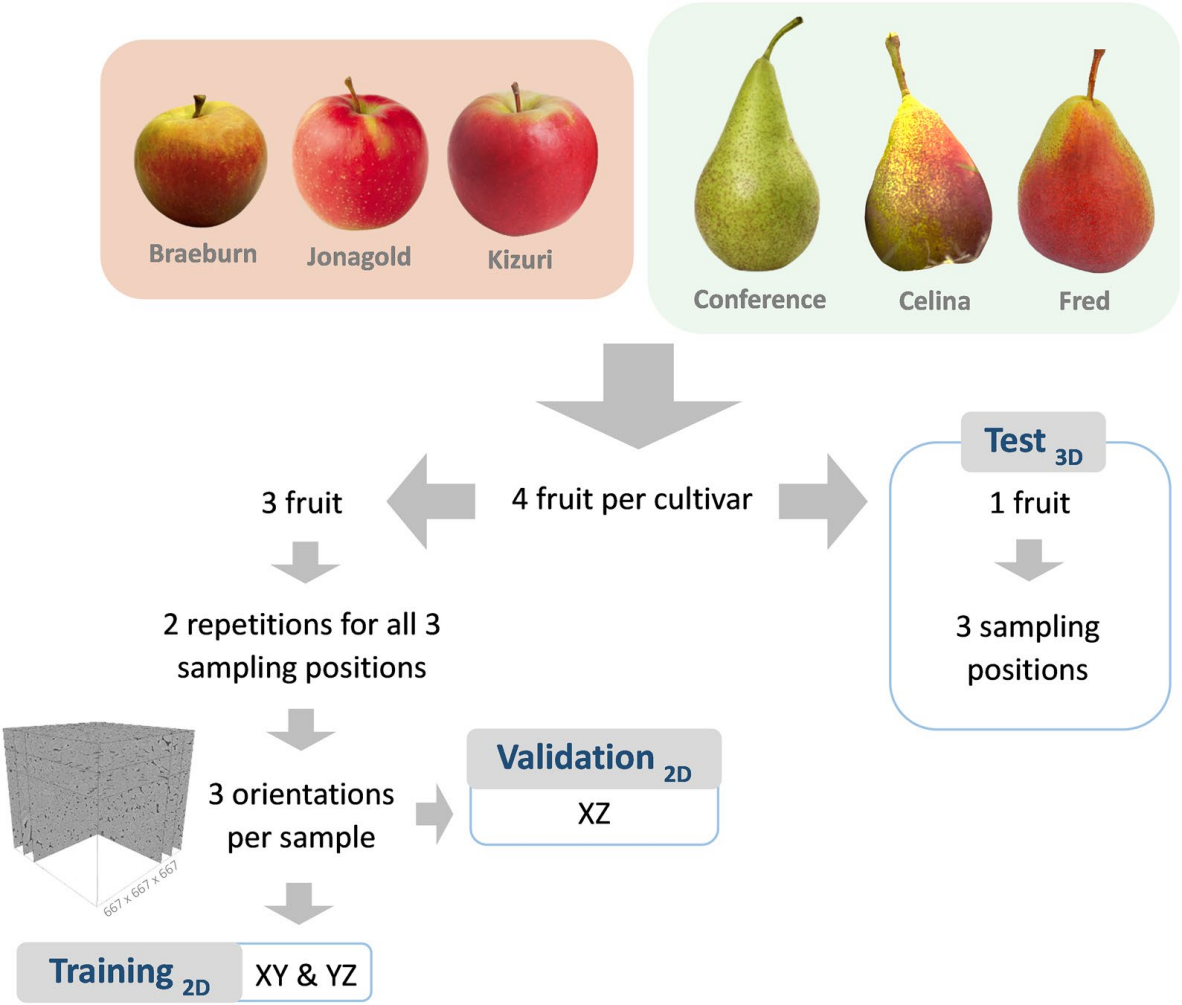
Data split into training, validation and test set

The dataset consisted of three repetitions per sampling position for each cultivar, thus 54 samples in total. Two repetitions per sampling position for each cultivar, 36 samples in total, were used in the training and validation set. The XY and YZ slices (667 × 667 pixels) were included in the training set and the XZ slices in the validation set. The test set included the VOIs (667 × 667 × 667 voxels) of the three sampling positions from one separate fruit for each cultivar, with 18 samples in total (Fig. 12).

Two experiments with different datasets for 2D training of the neural network were set up:

1. Training with a different number of slices per dataset
2. Training on different fruit datasets

Every tissue sample consisted of 667 2D slices in each orientation. To investigate whether the training would need all slices to perform proper cell segmentation, in the first experiment, seven training datasets were made by skipping XY and YZ slices in each dataset. Hereto, spacings of 8, 16, 32, 64, 128, 256 and 512 slices were considered, resulting in different amounts of slices in the training sets (Table 4). The XZ slices for the validation set were collected with 512 slice spacing, thus containing 36 images. For the second experiment, three training datasets were made using a spacing of 8 slices: apple and pear data separately, and combined. For the combined dataset only the first half of the slices in XY and YZ orientations were used to have a total amount of 5976 slices in each training set. The validation set of the first experiment was re-used in the second experiment.

**Table 4:** Spacings by which 2D slices were extracted resulting a different number of slices per training set

Slice spacing	Slices per sample	Slices in training set
8	83	5976
16	41	2952
32	20	1440
64	10	720
128	5	360
256	2	144
512	1	72

### 3D cell segmentation

Deep neural networks were trained for cell segmentation of the fruit tissue samples imaged with conventional X-ray micro-CT using the individual cell labels as GT data. The *Cellpose* network of Stringer et al. (2020), implemented by https://github.com/MouseLand/cellpose in the PyTorch framework in Python, was used.

The models of the two experiments described in the previous section were trained for 250 epochs with the RAdam optimizer (*β*_1_ = 0.95, *β*_2_ = 0.999) using a weight decay of 10^–5^ and *ϵ* of 10^–8^. The combined loss function consisted of the L2 loss for the horizontal and vertical gradient maps which were multiplied by a factor of five and a cross-entropy loss for the region of interest (ROI) map. Random rotation, scaling and flipping were used for data augmentation. Hyperparameter tuning was performed as described in Additional file 7 to then train the models with the best combination of hyperparameters and model architecture. An initial learning rate of 0.002 was decayed by a factor of 0.1 at epoch 200 and a batch size of 8 images was used. The first layer consisted of 48 feature maps and the network had a depth of 4 layers. The training and validation loss during training on a different number of slices per dataset and on different fruit datasets are show in Additional file 7: Figs. S1 and S2, respectively.

In the testing phase, the 3D extension framework of Stringer et al. (2020) was adopted. The 2D trained models were used to predict the ROI map along the horizontal and vertical gradient maps on all XY, YZ and XZ slices independently. The 3D ROI and vector gradients were then obtained by averaging. Additionally, the binary of the cell matrix retrieved from the conventional scans (see Additional file 4) was multiplied with the 3D ROI map. Then, the gradient tracking algorithm was ran to cluster the voxels to cell labels.

### Verification of segmentation accuracy

The DL based segmentation of the test set data was compared to the GT using the AJI. This evaluation metric was proposed by Kumar et al. [45] to evaluate nuclear segmentation results from microscopy images. The AJI penalizes both detection (object-level) and segmentation (px-level) errors as opposed to other metrics that focus on one type of error. First, for each combination of GT and segmented label, the Jaccard index (Intersection over Union metric) was calculated, defined as [46]:

1 $$J_{ij} = \frac{{\left| {G_{i} \cap S_{j} } \right|}}{{\left| {G_{i} \cup S_{j} } \right|}}$$

where $G_{i}$ and $S_{j}$ are the sets of voxels in GT label $i$ and segmented label $j$, respectively. Then, each GT label was matched with the segmented cell label that yielded the highest Jaccard index, with each segmented cell label only used once. Finally, using these pairs of GT and segmented cell labels, the AJI was calculated as follows:

2 $$AJI = \frac{{\sum\nolimits_{i = 1}^{N} {\left| {G_{i} \cap S_{M}^{i} } \right|} }}{{\sum\nolimits_{i = 1}^{N} {\left| {G_{i} \cup S_{M}^{i} } \right| + \sum\nolimits_{F \in U} {\left| {S_{F} } \right|} } }}$$

where $G_{i}$ is the $i^{th}$ GT label within the sample that contains $N$ cell labels. $S_{M}^{i}$ is the $M^{th}$ segmented cell label which has the largest Jaccard Index with $G_{i}$. In case no segmented cell label could be assigned to a GT label, the intersection term in the numerator is zero and the union term in the denominator is equal to $\left| {G_{i} } \right|$, penalizing false negatives. $U$ is the set of segmented cell labels that could not be assigned to a GT label and this term, therefore, penalizes false positives. This evaluation metric penalizes thus false positives, false negatives, under and over-segmentation of true positives. The AJI ranges between 0 and 1, with the first in case of no intersection between GT and the segmented sample and the latter for a perfect match. The calculation of the AJI was performed in Python 3.8 after adapting the implementation of Stringer et al. (2020) to 3D evaluation.

### Benchmark

The current state-of-the-art method for cell segmentation in micro-CT images of plant tissue relies on semi-automatic marker-based watershed segmentation and sieving as reported by Herremans et al. (2015). Therefore, as a benchmark to demonstrate progress made by the DL method, the marker-based watershed algorithm [47] was applied to the binary of the cell matrix in Avizo using marker extents of 0, 1, 2, 3 and 4 as shown in Additional file 2. The best marker extent for the benchmark was chosen according to the highest AJIs.

### Statistical analysis

All statistical analyses were done in RStudio with data visualization using ggplot2 [48] and ggpubr [49]. For the statistical analysis of the morphometric parameters, two-way ANOVA and post-hoc Tukey was used. In case of nonnormality or inhomogeneous variances, rank transformation of the data was performed [50]. To compare the segmentation results of the developed models, repeated-measures ANOVA and post hoc pairwise paired sample t-tests with Benjamini–Hochberg multiple testing correction method were performed on the nine test samples of each fruit type using the rstatix package [51]. A PLSR with full cross-validation was performed on standardized data using mdatools [44] to understand how the segmentation quality can be linked to the microstructure. The AJIs of the best model were used as response variable. For the predictor variables, only the morphometric parameters that could also be collected from conventional scans were considered.

### Supplementary Information

**Additional file 1: **Morphometric properties of individual cells and pores.

**Additional file 2: **Benchmark.

**Additional file 3: **Image registration.

**Additional file 4: **Cell matrix and pore labeling.

**Additional file 5: **Semi-automated cell segmentation workflow.

**Additional file 6: **Description of the 3D morphometric parameter.

**Additional file 7: **2D training of Cellpose network.

## Data Availability

The datasets used and analysed during the current study are available from the corresponding author on reasonable request.

## Abbreviations

AJI: Aggregated Jaccard Index
CCN: Convolutional neural network
DL: Deep learning
GT: Ground truth
IQR: Interquartile range
PLS-DA: Partial least squares discriminant analysis
LV: Latent variable
PLSR: Partial least squares regression
ROI: Region of interest
SSA: Specific surface area
VOI: Volume of interest
