# Supplementary material for: Automatic 3D cell segmentation of fruit parenchyma tissue from X-ray micro CT images using deep learning
Source: Plant Methods. 2024 Jan 19;20:12. doi: 10.1186/s13007-024-01137-y (PMC10799452; doi:10.1186/s13007-024-01137-y)
Supplement: Supplementary file 1 — Additional file 1: Morphometric properties of individual cells and pores. [file 13007_2024_1137_MOESM1_ESM.docx]

# Additional file 1. Morphometric properties of individual cells and pores

Table S1. Morphometric parameters of the cells of three tissue samples (mean ± SD) per cortex position and pome fruit cultivar.

| **Parameter** | **Cortex** | **‘Celina’** | **‘Conference’** | **‘Fred’** | **‘Braeburn’** | **‘Jonagold’** | **‘Kizuri’** |
| --- | --- | --- | --- | --- | --- | --- | --- |
| Length  [µm] | Inner | 213 ± 66^Bd^ | 201 ± 65^Bf^ | 207 ± 97^Ce^ | 326 ± 73^b^ | 353 ± 90^Ba^ | 276 ± 59^Bc^ |
|  | Middle | 237 ± 71^Ae^ | 219 ± 62^Af^ | 286 ± 119^Ad^ | 333 ± 80^b^ | 366 ± 87^Aa^ | 292 ± 57^Ac^ |
|  | Outer | 193 ± 72^Cf^ | 222 ± 71^Ae^ | 241 ± 110^Bd^ | 324 ± 76^b^ | 341 ± 71^Ba^ | 281 ± 57^Bc^ |
| Width  [µm] | Inner | 119 ± 35^Ad^ | 108 ± 27^Be^ | 90 ± 28^Cf^ | 177 ± 33^Bb^ | 184 ± 30^Ba^ | 168 ± 33^Cc^ |
|  | Middle | 121 ± 33^Ad^ | 122 ± 32^Ac^ | 111 ± 35^Ae^ | 173 ± 32^Bb^ | 190 ± 30^Aa^ | 187 ± 31^Aa^ |
|  | Outer | 107 ± 38^Be^ | 121 ± 33^Ad^ | 105 ± 35^Bf^ | 187 ± 39^Ab^ | 193 ± 34^Aa^ | 178 ± 33^Bc^ |
| Surface area  [10^3^ µm²] | Inner | 76 ± 38^Bd^ | 65 ± 30^Be^ | 55 ± 36^Cf^ | 162 ± 52^Ab^ | 179 ± 57^Ba^ | 133 ± 46^Cc^ |
|  | Middle | 82 ± 37^Ad^ | 79 ± 34^Ae^ | 89 ± 49^Ac^ | 159 ± 52^Bb^ | 190 ± 53^Aa^ | 156 ± 48^Ab^ |
|  | Outer | 64 ± 40^Cf^ | 80 ± 39^Ad^ | 74 ± 46^Be^ | 173 ± 62^Ab^ | 182 ± 56^Ba^ | 145 ± 49^Bc^ |
| Equivalent diameter [µm] | Inner | 137 ± 37^Bd^ | 127 ± 30^Be^ | 111 ± 35^Cf^ | 204 ± 36^Bb^ | 212 ± 34^Ba^ | 186 ± 35^Cc^ |
|  | Middle | 141 ± 35^Ac^ | 139 ± 33^Ac^ | 140 ± 43^Ac^ | 202 ± 36^Bb^ | 220 ± 31^Aa^ | 205 ± 33^Ab^ |
|  | Outer | 123 ± 41^Cf^ | 139 ± 36^Ad^ | 128 ± 42^Be^ | 213 ± 42^Ab^ | 216 ± 36^Ba^ | 196 ± 35^Bc^ |
| Volume  [10^6^ µm³] | Inner | 1.63 ± 1.20^Bd^ | 1.25 ± 0.82^Be^ | 0.94 ± 0.82^Cf^ | 4.82 ± 2.36^Bb^ | 5.39 ± 2.53^Ba^ | 3.74 ± 2.03^Cc^ |
|  | Middle | 1.74 ± 1.16^Ac^ | 1.63 ± 0.99^Ac^ | 1.81 ± 1.44^Ac^ | 4.70 ± 2.25^Bb^ | 5.92 ± 2.37^Aa^ | 4.84 ± 2.33^Ab^ |
|  | Outer | 1.30 ± 1.16^Cf^ | 1.67 ± 1.10^Ad^ | 1.45 ± 1.27^Be^ | 5.61 ± 2.88^Ab^ | 5.69 ± 2.58^Ba^ | 4.35 ± 2.30^Bc^ |
| Anisotropy | Inner | 0.68 ± 0.17^Bc^ | 0.70 ± 0.17^Ab^ | 0.76 ± 0.21^Ba^ | 0.70 ± 0.15^Bbc^ | 0.71 ± 0.15^Ab^ | 0.63 ± 0.16^Ad^ |
|  | Middle | 0.73 ± 0.17^Ab^ | 0.68 ± 0.18^Bd^ | 0.82 ± 0.19^Aa^ | 0.73 ± 0.14^Abc^ | 0.71 ± 0.15^Ac^ | 0.58 ± 0.16^Be^ |
|  | Outer | 0.68 ± 0.19^Bc^ | 0.70 ± 0.17^ABb^ | 0.77 ± 0.20^Ba^ | 0.67 ± 0.15^Cd^ | 0.68 ± 0.15^Bd^ | 0.59 ± 0.16^Be^ |
| Specific surface area [mm^-1^] | Inner | 57 ± 19^Bc^ | 61 ± 17^Ab^ | 74 ± 23^Aa^ | 37 ± 9^Ae^ | 36 ± 7^Af^ | 40 ± 10^Ad^ |
|  | Middle | 56 ± 18^Bb^ | 57 ± 18^Bb^ | 62 ± 22^Ca^ | 37 ± 9^Ac^ | 34 ± 6^Bd^ | 35 ± 7^Cd^ |
|  | Outer | 65 ± 23^Ab^ | 57 ± 19^Bc^ | 67 ± 24^Ba^ | 35 ± 10^Be^ | 35 ± 8^ABe^ | 37 ± 9^Bd^ |
| Length/width | Inner | 1.83 ± 0.48^Bd^ | 1.88 ± 0.52^Ac^ | 2.25 ± 0.80^Ba^ | 1.87 ± 0.42^Bc^ | 1.94 ± 0.48^Ab^ | 1.66 ± 0.33^Ae^ |
|  | Middle | 2.01 ± 0.57^Ab^ | 1.85 ± 0.52^Bc^ | 2.58 ± 0.89^Aa^ | 1.95 ± 0.47^Ab^ | 1.95 ± 0.50^Ab^ | 1.58 ± 0.27^Bd^ |
|  | Outer | 1.82 ± 0.49^Bc^ | 1.86 ± 0.49^ABb^ | 2.28 ± 0.83^Ba^ | 1.76 ± 0.35^Cc^ | 1.79 ± 0.35^Bbc^ | 1.60 ±0.28^Bd^ |
| Sphericity | Inner | 0.84 ± 0.06^Ab^ | 0.84 ± 0.07^Aab^ | 0.82 ± 0.10^Ad^ | 0.83 ± 0.06^Bc^ | 0.81 ± 0.06^Bd^ | 0.84 ± 0.06^Ba^ |
|  | Middle | 0.82 ± 0.08^Bc^ | 0.82 ± 0.08^Bbc^ | 0.78 ± 0.10^Cd^ | 0.83 ± 0.06^Bb^ | 0.82 ± 0.06^Ac^ | 0.86 ± 0.04^Aa^ |
|  | Outer | 0.85 ± 0.08^Ac^ | 0.82 ± 0.08^Bd^ | 0.80 ± 0.10^Be^ | 0.85 ± 0.05^Ab^ | 0.82 ± 0.05^Ad^ | 0.86 ± 0.05^Aa^ |

Different upper case characters in the same column for each parameter indicate significant differences (p < 0.05) at different cortex position for the same cultivar. Different lower case characters in the same row indicate significant differences (p < 0.05) among different cultivars for the same cortex position.

Table S2. Morphometric parameters of the pores of three tissue samples (mean ± SD) per cortex position and pome fruit cultivar.

| **Parameter** | **Cortex** | **‘Celina’** | **‘Conference’** | **‘Fred’** | **‘Braeburn’** | **‘Jonagold’** | **‘Kizuri’** |
| --- | --- | --- | --- | --- | --- | --- | --- |
| Length  [µm] | Inner | 65 ± 45^Ad^ | 42 ± 29^Ce^ | 45 ± 36^Ae^ | 167 ± 123^b^ | 180 ± 124^Ba^ | 135 ± 107^Cc^ |
|  | Middle | 62 ± 42^Bd^ | 44 ± 31^Be^ | 46 ± 47^Bf^ | 168 ± 113^c^ | 228 ± 134^Aa^ | 201 ± 129^Ab^ |
|  | Outer | 59 ± 43^Cb^ | 48 ± 38^Ac^ | 40 ± 34^Cd^ | 190 ± 131^a^ | 222 ± 168^Ba^ | 178 ± 125^Ba^ |
| Width  [µm] | Inner | 28 ± 18^Ad^ | 22 ± 13^Be^ | 17 ± 11^Bf^ | 74 ± 58^Bb^ | 80 ± 58^Ba^ | 67 ± 58^Cc^ |
|  | Middle | 25 ± 16^Bc^ | 23 ± 15^Ad^ | 19 ± 15^Ae^ | 73 ± 52^ABb^ | 101 ± 66^Aa^ | 105 ± 73^Aa^ |
|  | Outer | 23 ± 17^Cb^ | 24 ± 17^Bc^ | 18 ± 12^Ad^ | 93 ± 71^Aa^ | 115 ± 91^Ba^ | 91 ± 70^Ba^ |
| Surface area  [10^3^ µm²] | Inner | 4.31 ± 5.29^Ad^ | 2.72 ± 3.30^Be^ | 1.92 ± 2.54^Cf^ | 47 ± 68^b^ | 53 ± 68^Ba^ | 39 ± 61^Cc^ |
|  | Middle | 3.78 ± 4.41^Bd^ | 3.13 ± 3.83^Ae^ | 2.88 ± 4.87^Af^ | 43 ± 54^c^ | 78 ± 93^Aa^ | 79 ± 100^Ab^ |
|  | Outer | 3.42 ± 4.71^Cb^ | 3.48 ± 4.99^Ac^ | 2.28 ± 3.33^Bd^ | 69 ± 89^a^ | 109 ± 146^Ba^ | 64 ± 87^Ba^ |
| Equivalent diameter [µm] | Inner | 26 ± 14^Ad^ | 22 ± 12^Be^ | 18 ± 10^Bf^ | 78 ± 58^b^ | 84 ± 58^Ba^ | 69 ± 56^Cc^ |
|  | Middle | 25 ± 13^Bc^ | 23 ± 13^Ad^ | 21 ± 14^Ae^ | 78 ± 52^b^ | 106 ± 65^Aa^ | 105 ± 71^Aa^ |
|  | Outer | 23 ± 13^Cb^ | 23 ± 14^Bc^ | 20 ± 12^Ad^ | 95 ± 69^a^ | 115 ± 89^Ba^ | 92 ± 68^Ba^ |
| Volume  [10^3^ µm³] | Inner | 18 ± 31 ^Ad^ | 12 ± 19 ^Be^ | 7 ± 12 ^Bf^ | 767 ± 1640 ^b^ | 843 ± 1587 ^Ba^ | 620 ± 1373 ^Cc^ |
|  | Middle | 16 ± 26 ^Bc^ | 14 ± 23 ^Ad^ | 13 ± 29 ^Ae^ | 637 ± 1108 ^b^ | 1442 ± 2579 ^Aa^ | 1563 ± 2921 ^Aa^ |
|  | Outer | 14 ± 36^Cb^ | 16 ± 30 ^Bc^ | 9 ± 19 ^Ad^ | 1274 ± 2378 ^a^ | 2474 ± 4569 ^Ba^ | 1211 ±2370 ^Ba^ |
| Anisotropy | Inner | 0.81 ± 0.24^Cb^ | 0.68 ± 0.23^Be^ | 0.79 ± 0.33^Aa^ | 0.78 ± 0.22^Ac^ | 0.80 ± 0.20^Ac^ | 0.74 ± 0.23^d^ |
|  | Middle | 0.82 ± 0.24^Ba^ | 0.67 ± 0.23^Ce^ | 0.68 ± 0.28^Bd^ | 0.79 ± 0.21^Ab^ | 0.81 ± 0.17^Ab^ | 0.75 ± 0.20^c^ |
|  | Outer | 0.82 ± 0.26^Aa^ | 0.68 ± 0.25^Ac^ | 0.67 ± 0.27^Cc^ | 0.76 ± 0.21^Bb^ | 0.75 ± 0.20^Bb^ | 0.76 ± 0.21^b^ |
| Specific surface area [mm^-1^] | Inner | 411 ± 222^Cc^ | 400 ± 173^Bb^ | 534 ± 255^Aa^ | 193 ± 136^e^ | 176 ± 127^Af^ | 215 ± 140^Ad^ |
|  | Middle | 422 ± 219^Bb^ | 399 ± 181^Cc^ | 456 ± 190^Ba^ | 183 ± 130^d^ | 140 ± 111^Be^ | 152 ± 128^Ce^ |
|  | Outer | 455 ± 232^Ab^ | 407 ± 180^Ac^ | 451 ± 183^Ba^ | 173 ± 135^d^ | 162 ± 138^Ad^ | 176 ± 136^Bd^ |
| Length/width | Inner | 2.44 ± 1.10^Cb^ | 1.90 ± 0.49^Bd^ | 2.56 ± 1.27^Aa^ | 2.39 ± 0.85^Aa^ | 2.39 ± 0.83^Aa^ | 2.15 ± 0.65^Ac^ |
|  | Middle | 2.58 ± 1.23^Ba^ | 1.88 ± 0.51^Cc^ | 2.19 ± 0.93^Bb^ | 2.44 ± 0.89^Aa^ | 2.47 ± 0.95^Aa^ | 2.09 ± 0.66^Bb^ |
|  | Outer | 2.59 ± 1.22^Aa^ | 1.98 ± 0.63^Ae^ | 2.04 ± 0.75^Cd^ | 2.20 ± 0.73^Bb^ | 2.07 ± 0.57^Bc^ | 2.14 ± 0.64^Abc^ |
| Sphericity | Inner | 0.76 ± 0.18^Cc^ | 0.86 ± 0.13^Aa^ | 0.85 ± 0.21^Cb^ | 0.73 ± 0.15^Ad^ | 0.71 ± 0.14^Ae^ | 0.75 ± 0.15^Ac^ |
|  | Middle | 0.77 ± 0.18^Bc^ | 0.85 ± 0.14^Bb^ | 0.87 ± 0.16^Ba^ | 0.72 ± 0.14^ABd^ | 0.67 ± 0.12^Bf^ | 0.70 ± 0.13^Ce^ |
|  | Outer | 0.78 ± 0.19^Ac^ | 0.85 ± 0.15^Cb^ | 0.88 ± 0.14^Aa^ | 0.72 ± 0.15^Bd^ | 0.71 ± 0.15^Ad^ | 0.72 ± 0.14^Bd^ |

Different upper case characters in the same column for each parameter indicate significant differences (p < 0.05) at different cortex position for the same cultivar. Different lower case characters in the same row indicate significant differences (p < 0.05) among different cultivars for the same cortex position.
