# Supplementary material for: Automatic 3D cell segmentation of fruit parenchyma tissue from X-ray micro CT images using deep learning
Source: Plant Methods. 2024 Jan 19;20:12. doi: 10.1186/s13007-024-01137-y (PMC10799452; doi:10.1186/s13007-024-01137-y)
Supplement: Supplementary file 2 — Additional file 2: Benchmark. [file 13007_2024_1137_MOESM2_ESM.docx]

# Additional file 2. Benchmark

 Individual cell labels as a benchmark obtained by the marker-based watershed algorithm on the binary of the cell matrix using different marker extents with labels shown in a colour scale for cell volume.
