# Supplementary material for: Automatic 3D cell segmentation of fruit parenchyma tissue from X-ray micro CT images using deep learning
Source: Plant Methods. 2024 Jan 19;20:12. doi: 10.1186/s13007-024-01137-y (PMC10799452; doi:10.1186/s13007-024-01137-y)
Supplement: Supplementary file 3 — Additional file 3: Image registration. [file 13007_2024_1137_MOESM3_ESM.docx]

# Additional file 3. Image registration

Image registration of conventional and contrast-enhanced micro-CT images of a pear tissue sample. (A) Manual pre-alignment of the images followed by (B) image registration using indicated subsamples and (C) final corresponding volumes of interest of 667 x 667 x 667 voxels.
