# Supplementary material for: Automatic 3D cell segmentation of fruit parenchyma tissue from X-ray micro CT images using deep learning
Source: Plant Methods. 2024 Jan 19;20:12. doi: 10.1186/s13007-024-01137-y (PMC10799452; doi:10.1186/s13007-024-01137-y)
Supplement: Supplementary file 4 — Additional file 4: Cell matrix and pore labeling. [file 13007_2024_1137_MOESM4_ESM.docx]

# Additional file 4. Cell matrix and pore labelling

 Segmentation workflow of pear tissue micro-CT images to collect (A) binary cell matrix and (B) individual pore labels with labels shown in a colour scale for pore volume. ‘CHV’ = Calinski-Harabasz value.
