# Supplementary material for: Automatic 3D cell segmentation of fruit parenchyma tissue from X-ray micro CT images using deep learning
Source: Plant Methods. 2024 Jan 19;20:12. doi: 10.1186/s13007-024-01137-y (PMC10799452; doi:10.1186/s13007-024-01137-y)
Supplement: Supplementary file 5 — Additional file 5: Semi-automated cell segmentation workflow. [file 13007_2024_1137_MOESM5_ESM.docx]

# Additional file 5. Semi-automated cell segmentation workflow and validation

Fig. S1 Segmentation workflow of contrast-enhanced pear tissue micro-CT images to collect (A) individual cell labels with labels shown in a colour scale for cell volume, (B) vascular tissue and (C) stone cells. Manual steps are indicated in italic font.

The collection of ground truth (GT) labels, which is crucial for developing supervised deep learning-based models, is typically labor intensive and often requires human intervention [1–3]. If this were not the case, the desired task could be implemented more easily without the need of collecting lots of data to feed into a deep neural network. For instance segmentation, each individual object in the image must be annotated accurately, which is very labor intensive and especially for 3D images. Therefore, in this work, an approach with as little manual intervention as possible was developed. To collect the GT of individual cell labels, an X-ray imaging protocol performing conventional and successive contrast-enhanced scans on the same tissue samples was applied. In this procedure, the tissue can be damaged or deformed, making the subsequent image processing pipeline for the GT collection of the individual cells difficult or even impossible. As samples can turn out to be unusable because of the scanning procedure or unsuccessful image registration, more scans must be collected than intended to be used. The developed semi-automated cell segmentation workflow consists of multiple image processing steps and only one manual threshold required human intervention (Fig. S1).

For validation, the contrast-enhanced images and corresponding individual cell labels of ‘Celina’ and ‘Jonagold’ were sampled into 27 subsamples of 256 x 256 x 256 voxels with an overlap of 51 voxels for neighboring subsamples in MATLAB (Supplementary Fig. 2). One subsample without vascular tissue and stone cells was selected per sample for manual correction on the cell labels collected from the semi-automated cell segmentation workflow. These manual corrections, carried out in the segmentation editor of Avizo, involved cell label removal, addition and adjusting (Supplementary Fig. 3). The segmented cell labels from the workflow were evaluated by comparison to the manual corrected labels using the Aggregated Jaccard Index (AJI) proposed by Kumar et al. (2017). The AJI was calculated for eight subsamples of ‘Celina’ and nine of ‘Jonagold’ and the results are shown in Supplementary Fig. 4. The AJIs of ‘Celina’ and ‘Jonagold’ were 0.960 ± 0.026 and 0.944 ± 0.017, respectively.

/

667 px

256 px

//

//

//

/

/

Fig. S2 Subsampling grid of the 667 x 667 x 667 voxels into 27 volumes of 256 x 256 x 256 voxels.

**A**


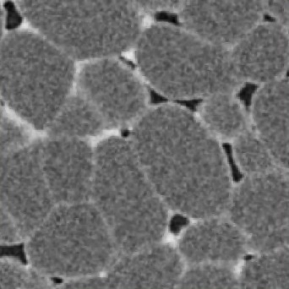

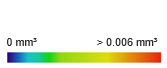


0

>0.006 mm³


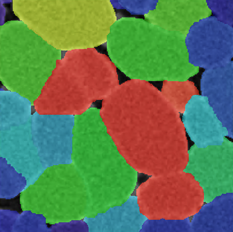

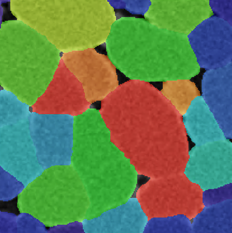


256 x 256 px

**B**

Fig. S3 (A) Individual cell labels from the semi-automated cell segmentation workflow of apple tissue micro-CT images and (B) manual corrected cell labels with labels shown in a colour scale for cell volume. Examples of corrections are indicated by the circles.


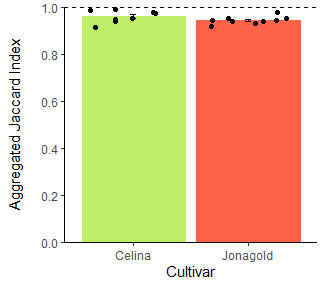


Fig. S4 Aggregated Jaccard Index with standard deviations for the error bars of segmented individual cell labels obtained with the developed semi-automated segmentation method from pear cultivar 'Celina' (n = 8) and apple cultivar 'Jonagold' (n = 9) tissue samples using manual corrected cell labels as ground-truth.

# References

1. Xu W, Yu G, Zare A, Zurweller B, Rowland D, Reyes-Cabrera J, et al. Overcoming Small Minirhizotron Datasets Using Transfer Learning. Comput Electron Agric. 2020;175.

2. Shen D, Wu G, Suk H-I. Deep learning in medical image analysis. Annu Rev Biomed Eng. 2017;19:221–48.

3. Tim Van De Looverbosch T, Vandenbussche B, Verboven P, Nicolaï B. Nondestructive high-throughput sugar beet seed analysis using X-ray CT and deep learning. Comput Electron Agric. 2021;200 May:submitted.

4. Kumar N, Verma R, Sharma S, Bhargava S, Vahadane A, Sethi A. A Dataset and a Technique for Generalized Nuclear Segmentation for Computational Pathology. IEEE Trans Med Imaging. 2017;36:1550–60.
