# Supplementary material for: Automatic 3D cell segmentation of fruit parenchyma tissue from X-ray micro CT images using deep learning
Source: Plant Methods. 2024 Jan 19;20:12. doi: 10.1186/s13007-024-01137-y (PMC10799452; doi:10.1186/s13007-024-01137-y)
Supplement: Supplementary file 6 — Additional file 6: Description of the 3D morphometric parameter. [file 13007_2024_1137_MOESM6_ESM.docx]

# Additional file 6. Description of the 3D morphometric parameters

Morphometric parameters on cell matrix, pore space, cell and pore level used to analyse the microstructure of apple and pear tissue.

| **Level** | **Parameter** | **Unit** | **Description** |
| --- | --- | --- | --- |
| **Cell matrix** | Cell density | mm^-3^ | Amount of cells per cubic millimeter |
|  | Stone cell density | mm^-3^ | Amount of stone cell clusters per cubic millimeter |
|  | Stone cell volume fraction | % | Stone cell volume divided by the total volume |
|  | Vascular tissue volume fraction | % | Vascular tissue volume divided by the total volume |
|  | Anisotropy | - | 1 minus the ratio of the smallest to the largest eigenvalue of the covariance matrix. Measures the deviation of an object from a spherical shape with 0 = perfect isotropy and 1 = complete anisotropy |
|  | Specific surface area | mm^-1^ | Surface area of the cell matrix divided by its volume |
| **Pore space** | Porosity | % | Pore space volume divided by the total volume |
|  | Pore density | mm^-S^ | Amount of pores per cubic millimeter |
|  | Anisotropy | - | 1 minus the ratio of the smallest to the largest eigenvalue of the covariance matrix. Measures the deviation of an object from a spherical shape with 0 = perfect isotropy and 1 = complete anisotropy |
|  | Specific surface area | mm^-1^ | Surface area of the pore space divided by its volume |
|  | Euler number | - | Euler-Poincaré number of the object which is an indicator of the connectivity of a 3D complex structure. The Euler number decreases with increasing connectivity [1] |
| **Cell/ pore** | Length | µm | Maximum of the Feret diameters measured over 31 angles |
|  | Width | µm | Minimum of the Feret diameters measured over 31 angles |
|  | Surface area | mm² | Surface area of the cell/pore |
|  | Equivalent diameter | µm | Diameter of a sphere with the same cell/pore volume |
|  | Volume | mm³ | Volume of the cell/pore |
|  | Anisotropy | - | 1 minus the ratio of the smallest to the largest eigenvalue of the covariance matrix. Measures the deviation of an object from a spherical shape with 0 = perfect isotropy and 1 = complete anisotropy |
|  | Specific surface area | mm^-1^ | Surface area of the cell/pore divided by its volume |
|  | Length/width | - | Length-to-width ratio |
|  | Sphericity | - | The surface area of a sphere with the same cell/pore volume divided by the cell/pore surface area [2] |

# References

1. Odgaard A, Gundersen HJ. Quantification of connectivity with special emphasis on 3D reconstractions. Bone. 1993;14:173–82.

2. Piovesan A, Achille C, Ameloot R, Nicolai B, Verboven P. Pore network model for permeability characterization of three-dimensionally-printed porous materials for passive microfluidics. Phys Rev E. 2019;99:1–13.
