# Supplementary material for: Automatic 3D cell segmentation of fruit parenchyma tissue from X-ray micro CT images using deep learning
Source: Plant Methods. 2024 Jan 19;20:12. doi: 10.1186/s13007-024-01137-y (PMC10799452; doi:10.1186/s13007-024-01137-y)
Supplement: Supplementary file 7 — Additional file 7: 2D training of Cellpose network. [file 13007_2024_1137_MOESM7_ESM.docx]

# Additional file 7. 2D training of Cellpose network

Hyperparameter tuning was performed to subsequently train the deep neural networks for cell segmentation of the fruit tissue samples. Hereto, different combinations of hyperparameters and architectural changes were evaluated for the dataset with a spacing of 64 slices. Learning rates (LRs) of 0.4, 0.2, 0.02, 0.002 and 0.0002 were tested in a fixed manner as well as in a learning rate schedule in which these LRs were decayed by factor 0.1 at epoch 200. Additionally, batch sizes of 8, 16 and 32 images were tested. Finally, 32 or 48 feature maps in the first layer in combination with the depth of 4 or 5 layers were evaluated.

When training for more than 250 epochs, the training loss kept decreasing while the validation loss started to increase. To prevent overfitting, the training was stopped at 250 epochs. The best results were obtained with an initial LR of 0.002 that was decayed by a factor of 0.1 at epoch 200 and a batch size of 8 images. Additionally, 48 feature maps in the first layer and a depth of 4 layers resulted in the lowest validation loss.

The aforementioned best combination of hyperparameters and model architecture that resulted in the smallest loss on the validation set were used to train the different models from the two experiments in this study. The training and validation loss during training on datasets collected using different slice spacings is presented in Supplementary Fig. 1. With decreasing slice spacing, and thus increasing amount of images in the training set, the loss curves of both the training and validation data were lower. The training and validation loss during training on apple and pear data separately and combined are shown in Supplementary Fig. 2. When trained with apple data, the loss was lower compared to a training with solely pear data. The training loss of the combined model was in between the loss of the specialist models, but the validation loss was the lowest as the validation set consisted of data from both apple and pear fruit.


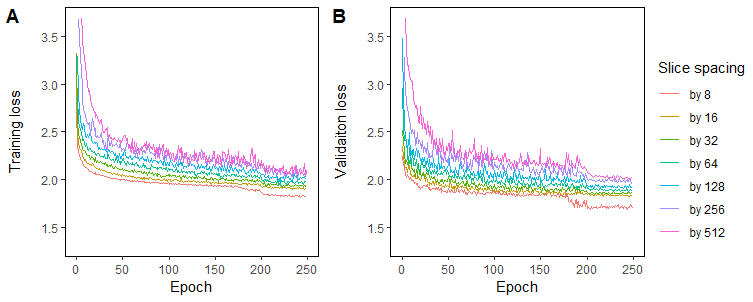


Fig. S1 (A) Training and (B) validation loss during training on the datasets obtained with different slice spacings.


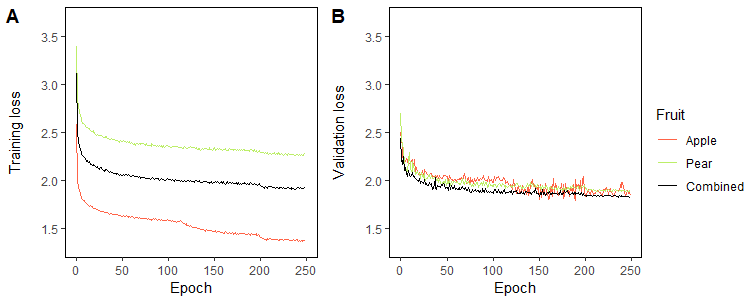


Fig. S2 (A) Training and (B) validation loss during training on different fruit datasets.
